# The “Bloodless” Blood Test: Intradermal Prick Nanoelectronics for the Blood Extraction-Free Multiplex Detection of Protein Biomarkers

**DOI:** 10.1021/acsnano.2c01793

**Published:** 2022-08-25

**Authors:** Nimrod Harpak, Ella Borberg, Adva Raz, Fernando Patolsky

**Affiliations:** † School of Chemistry, Faculty of Exact Sciences, Tel Aviv University, Tel Aviv69978, Israel; ‡ Department of Materials Science and Engineering, the Iby and Aladar Fleischman Faculty of Engineering, Tel Aviv University, Tel Aviv69978, Israel

**Keywords:** Detection, Biomolecules, Nanosensors, Nanobioelectronics, Biomarkers

## Abstract

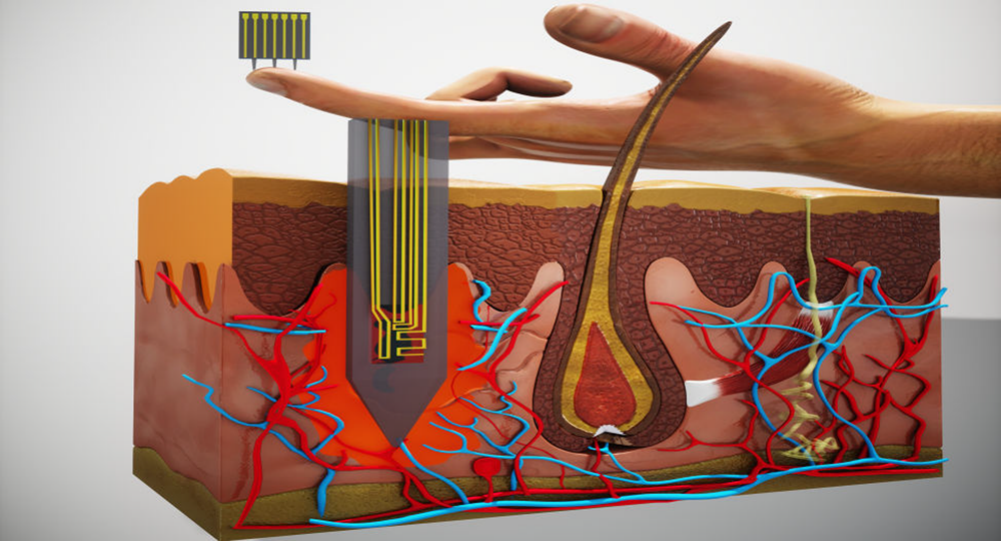

Protein biomarkers’ detection is of utmost importance for preventive medicine and early detection of illnesses. Today, their detection relies entirely on clinical tests consisting of painful, invasive extraction of large volumes of venous blood; time-consuming postextraction sample manipulation procedures; and mostly label-based complex detection approaches. Here, we report on a point-of-care (POC) diagnosis paradigm based on the application of intradermal finger prick-based electronic nanosensors arrays for protein biomarkers’ direct detection and quantification down to the sub-pM range, without the need for blood extraction and sample manipulation steps. The nanobioelectronic array performs biomarker sensing by a rapid intradermal prick-based sampling of proteins biomarkers directly from the capillary blood pool accumulating at the site of the microneedle puncture, requiring only 2 min and less than one microliter of a blood sample for a complete analysis. A 1 mm long microneedle element was optimal in allowing for pain-free dermal sampling with a 100% success rate of reaching and rupturing dermis capillaries. Current common micromachining processes and top-down fabrication techniques allow the nanobioelectronic sensor arrays to provide accurate and reliable clinical diagnostic results using multiple sensing elements in each microneedle and all-in-one direct and label-free multiplex biomarkers detection. Preliminary successful clinical studies performed on human volunteers demonstrated the ability of our intradermal, in-skin, blood extraction-free detection platform to accurately detect protein biomarkers as a plausible POC detection for future replacement of today’s invasive clinical blood tests. This approach can be readily extended in the future to detect other clinically relevant circulating biomarkers, such as miRNAs, free-DNAs, exosomes, and small metabolites.

## Introduction

Detection of clinical biomarkers is of enormous importance, particularly in the field of medicine, as they can provide critical data regarding an individual’s medical condition and may assist, by proper early diagnosis, in managing diseases, and preventing mortalities. However, modern-day medical diagnosis heavily relies on blood tests as the primary indicator for human health, as blood contains tens of thousands of proteins, biomarkers, and other biological species. Most of today’s processes for reliable detection and quantification of biomarkers require time-consuming and complex separation methods of the bodily fluid in order to separate blood cells and other interrupting constituents.^1−4^ Such sample preparation steps often lead to reduced sensitivities and a lack of reliability in specific assays, along with the incapability to perform point-of-care (POC) analysis.^5−7^

The current widespread blood tests rely solely on painful and invasive venous blood extraction of several tens of milliliters in volume. Despite the fact that it is currently the preferred diagnostic approach, some of the extracted biosamples are eventually discarded after centrifugation due to technical factors related to sample handling, transportation, storage conditions, and postextraction manipulation.^8,9^ As the world shifts its attention toward POC medical devices, which will ultimately result in simpler methods of analysis and diagnosis, more reliable and accurate methods are required to detect diagnostic biomarkers. POC testing is defined as medical diagnostic assays at or near the point of care—that is, at the time and place of patient care. This contrasts with the historical arrangement in which testing was wholly confined to central medical laboratories, which required sending specimens away from the point of care, then waiting hours or days to reach results, during which time care must continue without the desired information. Additionally, measuring multiple bioanalytes simultaneously in the same sample is very desirable, allowing a rapid, low-cost, and reliable quantification. Thus, multiplexed POC testing has become critically important for medical diagnostics. In this context nowadays, capillary blood-based diagnosis approaches are being vigorously investigated, as they may ultimately offer less invasive, faster, cheaper, and more straightforward means for POC diagnosis. However, as many currently developed sensing strategies attempt to realize successful diagnosis based on capillary blood sample analysis,^10,11^ numerous problems arise from the minimal volumes needed to be extracted and subsequently required for effective analysis: uncontrollable detrimental effects experienced by the blood samples upon extraction and postextraction manipulation steps, normally occurring before reaching the final sensing phase, such as clotting and hemolysis;^12,13^ substantial limitations of postextraction sample manipulation steps originating from the tiny volume of extracted samples, in the range of few microliters, further preventing the application of centrifugation and additional required procedures; and the final incapability to perform multiplexed biomarkers analysis on these small volume samples. All these factors lead to significant analytical artifacts impeding diagnosis. Hence, a new paradigm is strongly required to quantitatively sample and analyze multiple clinical biomarkers of interest directly from the patient’s capillary blood confined to the intradermal space *in vivo*, unrestricted to current diagnostic technologies requirements of blood samples extraction and postextraction storage, transportation, and manipulation steps.

In this regard, microneedle-based systems have been recently shown to be one of the most appealing concepts for *in vivo* intradermal applications. Because of their size, these systems were demonstrated to be minimally invasive easy-to-use platforms, where no severe tissue damage is observed by their long-term use.^14−17^ Most of these systems’ applications have focused on drug delivery,^18−20^ liquid biosamples extraction for *ex situ* analysis,^21,22^ and glucose levels monitoring in diabetic individuals.^23,24^ Currently reported microneedle-based sensing platforms are based on complex nonscalable fabrication procedures, often limiting the resulting devices’ reliability, accuracy, and real-world applicability.^25−28^ Furthermore, all reported studies focused on the real-time intradermal detection of small molecular species, mostly glucose. No report exists on the direct *in vivo* detection of protein biomarkers from the intradermal space. Nanowires^29−33^ have been shown to be a versatile substrate for the fabrication of devices in a broad range of applications such as electronics,^34,35^ optics,^36^ biosciences,^37,38^ medical diagnosis,^24^ and energy storage.^39−41^ More specifically, silicon nanowire-based field-effect transistors (SiNW-FET) were recognized in the last two decades as plausible candidates for label-free, ultrasensitive biosensing devices,^42−46^ allowing biomarkers detection in the deep sub-pM concentration range, thus covering the clinically relevant biofluid concentrations of most biomarkers of interest. Unfortunately, their intrinsic low limit of detection is achievable only under low-ionic strength conditions due to Debye length screening limitations imposed by the high ionic content of the body fluid under analysis, with ionic strengths higher than 150 mM and Debye length of ca. 1 nm. This significant handicapping limitation prohibits any practical applications of SiNW-FET devices for sensing unprocessed complex biological fluids. In recent years, successful attempts to overcome the Debye screening length limitation were presented, utilizing the “delayed-dissociation” of surface-bound antigen–antibody pairs^47,48^ and additional approaches.^49,50^ These later studies allowed the sensing of bioanalytes in postextraction processed whole blood samples (e.g., serum and plasma), as well as directly from unprocessed whole plasma samples in the former case, practically without limiting the analytes that can be quantitatively measured and depending on the surface-modified antibody of choice. However, while these methods allow exploitation of the complete intrinsic sensitivity of the SiNW-based nanoelectronic devices, still, the extraction of blood samples is required, like in all widespread blood-extraction-based clinical diagnostic platforms.

In light of the above, an appealing concept would be to implement and combine SiNW-FET devices with a microneedle-based system. Such a system would be able to answer the many desires arising for a complete biosensing apparatus: (i) capability to perform highly sensitive sensing of biomolecules, down to the sub-pM range, directly from blood; (ii) minimally invasive probing; (iii) rapid measurement times and reliable results for a complete POC device; (iv) multiplexed detection of various biomolecules on the same device; and (v) scalable fabrication. The microneedle embedded nanosensor arrays are created by conventional 2D fabrication procedures integrated to fabricate a functional intradermal probing platform. The sensing microneedle probe is capable of impaling the outer dermal layer down to a depth dictated by the microneedle length, rupturing capillaries, and forming a blood pool at the puncture site. A few seconds long intradermal probing of the blood pool by the nanosensor array at the tip of the microneedle element, followed by the *ex vivo* detection step, leads to the accurate and quantitative relative biomarker of interest.

Here, we present a paradigm of a fully integrated microneedle-embedded SiNW-FET devices’ array capable of performing POC rapid label-free sensing of multiple protein biomarkers by a minimally invasive, pain-free method directly from the intradermal space without the requirement for blood extraction and manipulation steps. The proposed fabrication workflow allows for devices’ redundancy and multiplexed detection, providing reliable results and multibiomarker detection capabilities by the same sensing platform. We show that by using our multifunctional sensing microneedle elements, protein biomarkers’ detection can be successfully performed directly from the intradermal tiny submicroliter capillary blood pools filling the impalement sites resulting from the dermal penetration of the microneedle elements. This work demonstrates diagnosis paradigm, based on the application of microneedle-embedded nanosensors arrays for the blood extraction-free direct intradermal capillary detection of protein biomarkers with a sub-pM sensitivity for all tested species (i.e., below 0.03 ng/mL). This diagnostic platform holds the future potential to replace the current painful and invasive diagnostic approaches based on blood extraction and manipulation procedures, dominating today’s medical blood tests, thus providing a simple POC device for the intradermal capillary rapid and accurate detection of protein biomarkers of interest.

## Results

**Figure 1:**
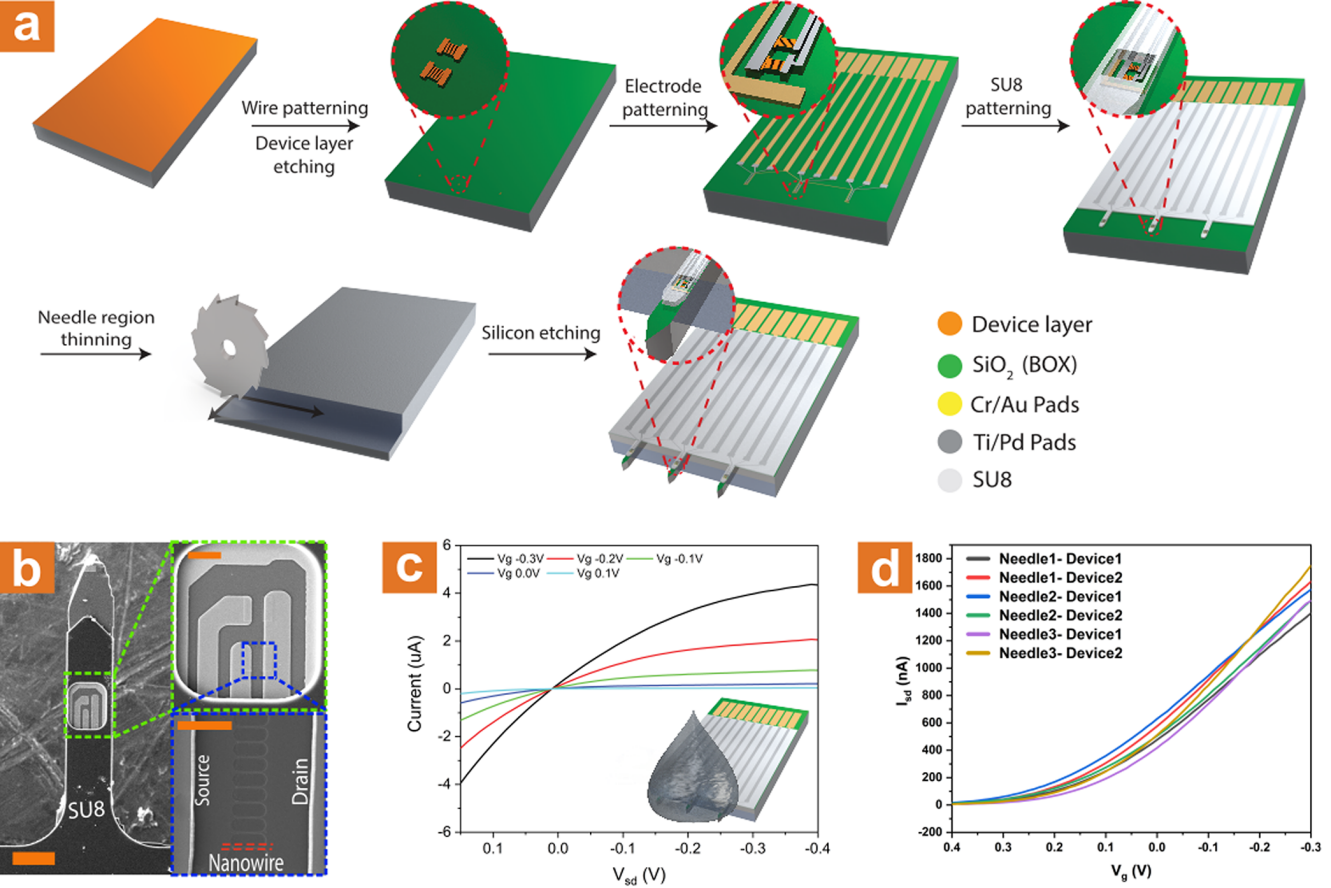
Fabrication and characterization of the SiNW-FET-based microneedle array sensor. (a) Schematic illustration of the top-down fabrication process. (b) SEM images of the fabricated needles; the needles are 150 μm in width and ca. 250 μm in thickness (scale bar: 125 μm). The green inset shows the opening of the SU-8 layer that forms the device window (scale bar: 25 μm). The blue inset shows a close-up image on one of the two devices inside the device window. The source-drain pads lie on pads fabricated from the device layer for better contact and surface area. The nanowires are a part of the device layer, laying on the buried oxide, and are 125 nm in width and 75 nm high (scale bar: 5 μm). (c) Electrical characterization of a representative device. The source-drain voltage was swept between −0.4 and 0.15 V, and the gate was kept constant at −0.3 V (black curve), −0.2 V (red curve), −0.1 V (green curve), 0 V (blue curve), and 0.1 V (light blue curve). Inset illustrates how the measurement was made, mimicking the *ex vivo* experiments as close as possible. (d) Transconductance measurements of five individual devices on the same microneedle FET. Vsd was kept constant on 0.1 V while the gate was swept between (−0.3) V to 0.4 V.

Intradermal probing requires careful planning of the sensing device, as some crucial elements are required to safeguard the entire device during skin impalement. Silicon-on-insulator (SOI) based devices have been on the rise in the past decade as an alternative to the common bottom-up vapor–liquid–solid (VLS) approach.^51−53^ SOI-based devices exhibit greater reproducibility, lower variability between devices, and can be fabricated using large-scale integration techniques, which enable complex designs to be executed very simply.^54^ The robust fabrication process of the microneedle-embedded SiNW-FET device is depicted in Figure 1a. An ultrathin device layer of 75 nm silicon-on-insulator (SOI) was selected, with a buried oxide (BOX) thickness of 400 nm. The initial thickness of the dies was 750 μm, in order to maintain the structural integrity of the whole microneedle-embedded device. Once the nanowires were patterned and formed, and the electrodes were fabricated using standard UV lithography and metal evaporation steps, a SU-8 chemically protecting layer was formed. It should be noted that once the nanowires are formed, no plasma processes were conducted in order to prevent the severe ion damage that will substantially lower the conductivity of the resulting devices.^55^ The SU-8 layer, which was patterned to leave open access to the sensing devices in the form of a 150 μm × 130 μm pool structure, is of crucial importance to the functional design of the microneedle-embedded SiNW FET elements. Beyond the potential contamination faced by the nanowires-based devices when impaling the skin, scrubbing of the sensing elements by the intradermal layers will mechanically abrase and remove the covalently attached molecular biorecognition layer upon impaling into the skin. Therefore, the heightening of the surface from the nanowires-based devices, by the SU-8 layer, is an essential and key element in the successful execution of extraction-free, intradermal protein detection. Once the SU-8 layer is formed, mechanical thinning of the needle region is conducted. The nanowire elements based on the SOI device layer are extremely prone to ion damage, which will ultimately result in loss of conductivity. Therefore, as part of the elaborate fabrication scheme, the time required to etch the final structure into the microneedle elements, using deep reactive ion etching (DRIE), is greatly reduced by the mechanical thinning. The final microneedle-embedded sensors can be seen in the SEM image provided in Figure 1b. The final needle structure possesses a sharp tip, allowing simple impalement of the skin layers. The final structure is ca. 150 μm wide and 250 μm thick. The SU-8 layer formed is approximately 5 μm thick, not impairing the ability of the needle to penetrate the skin. For the purpose of this manuscript, two sensing elements are fabricated on each needle as can be seen in the insets. In this context, it should be noted that the amount of sensing elements for each needle is limited only by the physical size of the desired needle; therefore, numerous devices can be directly fabricated, allowing higher sensing redundancy. The robust fabrication scheme is carefully designed to provide various desired properties in a single on-chip POC device: (i) independent sensing capabilities for each needle, allowing multiplexed detection of various analytes through different chemical modifications; (ii) multiple sensors in each individual needle for redundancy purposes, allowing reliable and accurate diagnostic results; and (iii) safekeeping of the sensing region during skin impalement. The ability to fabricate the whole device using common micromachining and lithography tools allows the number of needles, devices, length, and shape to be easily varied.

Then, the resulting microneedle-embedded SiNW FET devices were electrically characterized using a probe station. Electrical *I*–*V* measurements are shown in Figure 1c. Using a top-gate characterization method, as illustrated in the inset, the device’s electrical characteristics exhibit a p-type behavior, undamaged by the DRIE process used to create the final microneedle structure. Transconductance measurements, depicted in Figure 1d, show a minor 8.5% variability between different devices.

**Figure 2:**
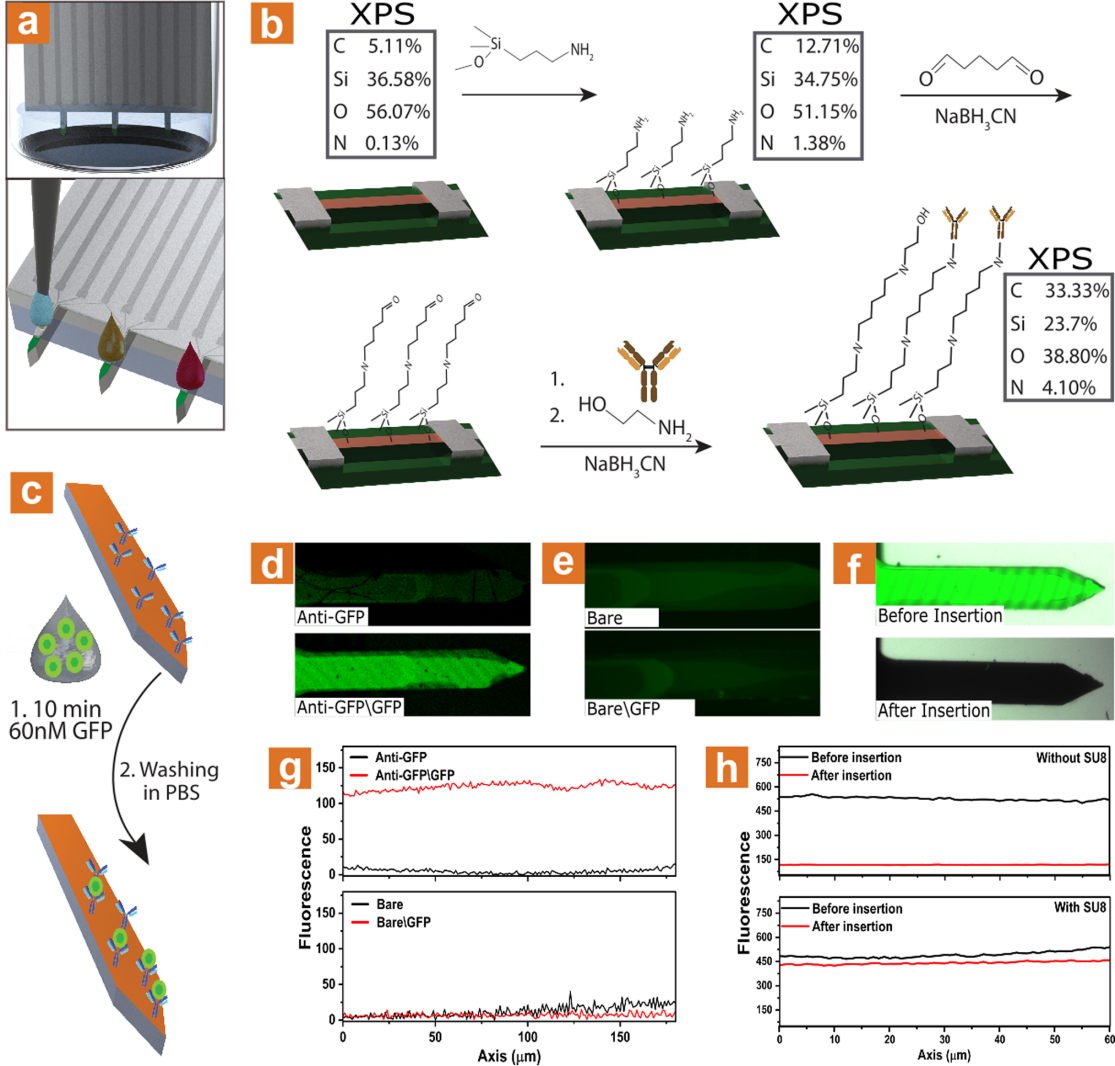
Surface modification process. (a) Illustration of modification. Top: microneedles dipped in 150–200 μL, bottom: each needle drop-cast using a microspotter. (b) Schematic illustration of the chemical modification process, with XPS results of different stages of the modification. (c) Schematic illustration of GFP binding to its antibody to test the modification process. (d) Fluorescence microscopy images results of GFP binding to needle after chemical immobilization of GFP-antibody. The needle is shown before (top) and after (bottom) soaking for 10 min in 60 nM GFP. (e) Fluorescence microscopy images results of GFP binding to bare needle. The needle is shown before (top) and after (bottom) soaking for 10 min in 60 nM GFP. (f) Fluorescence microscopy images of Alexa488 chemically immobilized to needles without SU-8 window before (top) and after (bottom) insertion to PDMS. (g) Fluorescence intensity of needles modified before (black) and after (red) soaking for 10 min in 60 nM GFP. Top: with GFP-antibody, bottom: bare needle, respectively, correlating to (d) and (e). (h) Fluorescence intensity of Alexa488 chemically immobilized to needles before (black) and after (red) insertion to PDMS. Without (top) and with SU-8 window (bottom).

Monitoring different proteins’ biomarkers can be crucial for the early detection of many diseases and medical conditions. What usually requires an invasive, painful process of drawing a few milliliters of venous blood for diagnosis can be practically avoided using minimal amounts of capillary blood via rapid antibody–antigen binding followed by electrical measuring. We modified the microneedle sensing surface with an anti-PSA antibody as a proof-of-concept. Elevated prostate-specific antigen level (PSA) is known to be a biomarker for prostate cancer, considered healthy up to 4 ng/mL (∼120 pM).^56,57^ Therefore, direct detection of blood-PSA can provide a significant measure for an individual’s health without having to extract blood in an invasive and painful manner. As illustrated in Figure 2a, the chemical modification can be conducted in two ways—either submerging the needles in 150–200 μL antibody solution or by using a microspotting system to dispense small volumes of antibody modification solution on each needle individually, allowing for easy multiplexing of the device, as illustrated at the bottom of Figure 2a.

The modification process is schematically illustrated in Figure 2b. First, 3-aminopropyldimethylethoxysilane (APDMES) was applied in an inert environment by dipping the needles in the pure silane derivative solution for 1 h, followed by a thorough wash in toluene and IPA, and placed on a heating plate at 70 °C for 30 min in order to evaporate the solvents fully. Then, the needles were dipped in a 200 μL of 10% glutaraldehyde solution in phosphate buffer containing 50 mg of cyanoborohydride for 1 h and washed thoroughly in IPA and DIW. The needles were then modified with a 30 μg/mL anti-PSA solution in phosphate buffer with 50 mg of cyanoborohydride at 4 °C. The blocking of unreacted aldehyde surface groups was performed via dipping the needles for 2 h in 200 μL of ethanolamine solution (150 μL in 20 mL phosphate buffer containing 50 mg of cyanoborohydride).

X-ray photoelectron spectroscopy (XPS) analysis results of the different modification steps are shown in Figure 2b (marked boxes). Once the amino-silane derivative APDMES is covalently attached, a rise in carbon and nitrogen atomic content is measured. As expected, a significant increase in carbon and nitrogen content occurs once the glutaraldehyde and antibody molecules are attached to the sensing surface. These XPS results demonstrate the successful modification of the sensing device surface with the receptor antibody molecules. Full XPS spectra are shown in Figure S2.

In order to further verify the chemical modification, fluorescence microscopy experiments were conducted. Instead of an anti-PSA antibody, anti-GFP (Green Fluorescent Protein) was modified onto the surface of a bare microneedle. The SU-8 protection layer was not used in this case since the high autofluorescence effect of SU-8 prevents proper fluorescence measurements. The microneedles were then dipped in a 60 nM GFP solution for 10 min, as illustrated in Figure 2c, and further washed in phosphate buffer saline (PBS). It can be clearly seen, Figure 2d, that a 10 min incubation leads to an apparent increase in the fluorescence intensity measured as a result of the specific binding of GFP to the surface of the antibody-modified microneedle element. Six different areas were tested along the needle length axis, marked with dots on Figure 2d, and fluorescence intensity values were normalized using the average intensity value measured on the chemically unreacted microneedle (not exposed to GFP). The results are plotted in Figure 2e, showing a ca. 600% increase in fluorescence intensity. Therefore, it can be concluded that the microneedle sensing surface was successfully modified with the antibody receptor molecules. To further verify the modification process, electrochemical impedance spectroscopy (EIS) measurements were performed. The results are shown in Figure S3. The measurements were performed using a three-electrode system (silicon piece as working electrode, platinum mesh as counter electrode and Ag/AgCl as a reference electrode) submerged in 0.01× PBS solution, under 20 mV amplitude at 0.1 V (this potential was chosen as no redox occurs in this point). As the modification process continues, a clear increase in the charge transfer resistance is seen. Furthermore, once incubated in protein for 10 min after antibody modification, a major increase in the charge transfer is observed.^58^

The fundamental importance of the SU-8 protective layer integrated into the core design of the microneedle-embedded sensors is shown in Figure 2f. A needle chemically modified with the fluorophore Alexa-488 is shown before (top) and after (bottom) its mechanical insertion into a polydimethylsiloxane (PDMS) skin-mimicking slab. The absence of a SU-8 protection layer leads to complete removal of the antibody biorecognition layer upon insertion of the microneedle elements into this slab and will not further allow the desired successful application of the chemically modified microneedle devices in the intradermal detection of biomarkers. Notably, no measurable difference was measured when performing the same experiment with chemically modified microneedle elements containing the SU-8 protective layer, as shown in Figure 2g. These results can also be verified using EIS spectroscopy as shown in Figure S3. After insertion of the silicon to PDMS (after incubation in protein), a major decrease in the charge transfer resistance can be seen. These results highlight the critical importance of the SU-8 chemistry-protective layer, which allows for microneedle transdermal penetration without compromising either the electronic devices’ performance or their chemical modification integrity.

**Figure 3:**
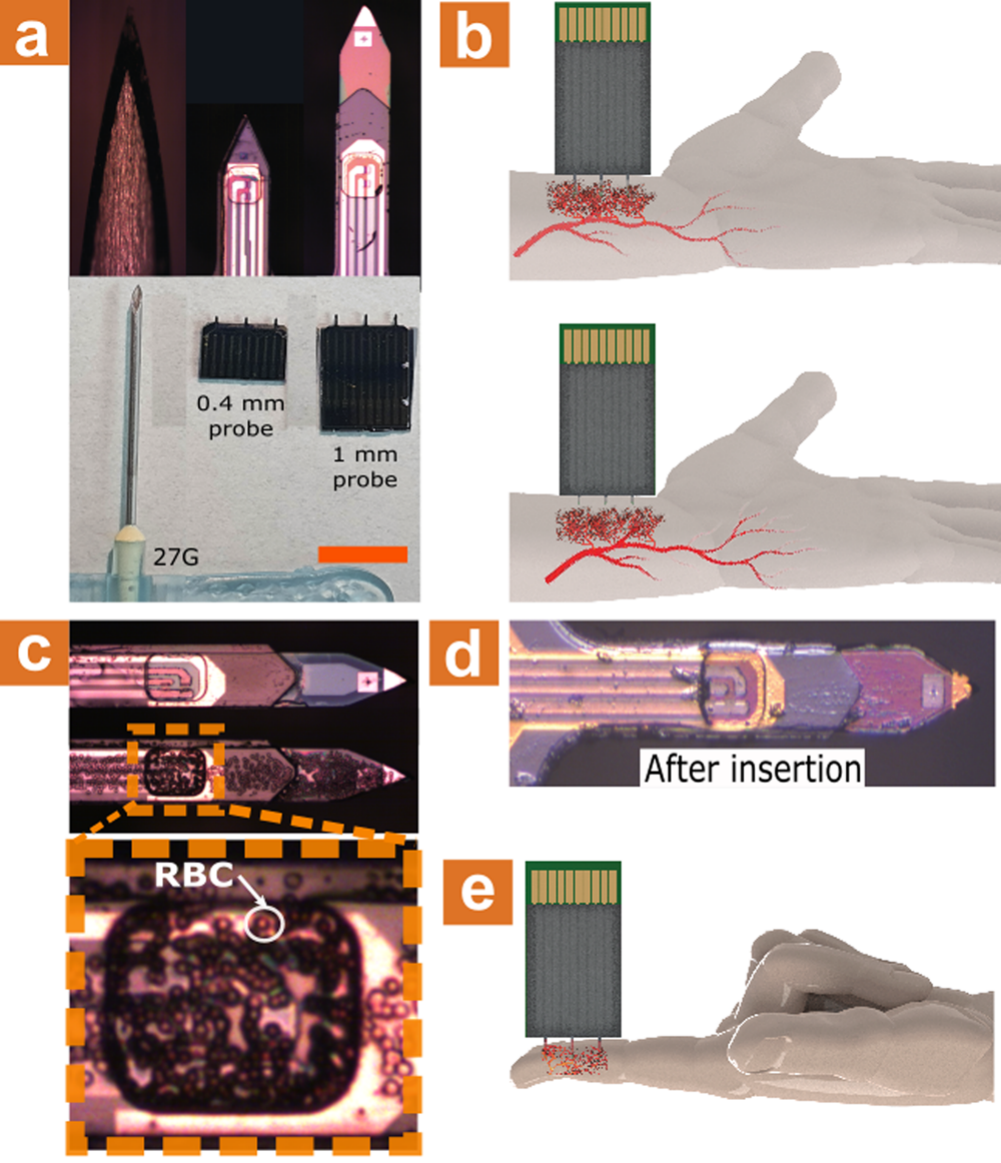
Microneedle array dimensions and blood contact. (a) Optical image of comparison in size between common 27G needle for venous blood extraction and the proposed microneedle array sensors. Two types of fabricated microneedles length are shown −400 μm and 1 mm. Scale bar: 5 mm. (b) Schematic illustration of microneedle insertion to the forearm. 1 mm microneedle should reach the blood capillaries in the dermis, while 400 μm needles will not reach as effectively. (c) Optical images showing the microneedle before (top) and after (bottom) a blood droplet was placed on the microneedle. Orange inset shows blood is clearly able to enter the SU-8 window. (d) Optical image showing the microneedle after insertion to the skin. (e) Schematic illustration of a different possible location for protein detection in the blood. The microneedle array can be used in the finger without or with prior pricking.

In order to prepare for *in vivo* sensing experiments, where protein biomarkers in capillary blood are captured directly at the nanosensors sites, without the requirement of extraction of bodily fluids, the proper length of microneedle elements should be chosen. Microneedles in the length of 300–500 μm were previously shown to enable *in vivo* transdermal monitoring of glucose levels in interstitial fluid (ISF); however, this insertion depth range does not allow microneedle elements to reach and rupture intradermal blood capillaries networks.^24^ Therefore, longer microneedles are needed to fully penetrate and rupture the dermal layers and reach capillary depth for the subsequent capillary blood protein biomarkers detection. Figure 3a shows a comparison of different needles–a 27G needle used in common venous blood extraction procedures, and representative 400 μm-long and 1 mm-long microneedle-embedded SiNW-FET arrays. In today’s phlebotomy practices, a needle is inserted up to a few millimeters for the purpose of drawing blood from a vein in the arm. Successful execution of protein biomarkers sensing by our microneedle-based system would reduce the need for such invasive procedures and would hold the potential to eventually replace these invasive diagnosis means by a minimally invasive and unpainful procedure. Figure 3b shows a schematic illustration of the difference between 400 μm and 1 mm microneedle apparatus. The subcutaneous capillary layer in the forearm and fingers’ tip is found to be approximately 0.6–1.5 mm in depth,^59^ while the epidermis layer is a few hundred micrometers thick.^60^ Therefore, a 1 mm needle is expected to penetrate the dermal layer and rupture blood capillaries smoothly in a nonpainful way.^61^ Diagnostically, capillary blood protein biomarkers’ levels have been shown to correlate well with those of venous blood,^62^ meaning that our minimally invasive, pain-free procedure by the microneedle-embedded nanosensors platform could represent a promising alternative to the currently widespread venous blood extraction-based diagnostics approaches.

Figure 3c shows optical microscope images of a microneedle element before and after its contact with a blood droplet. As seen in the inset, the blood droplet, as evidenced by the presence of red blood cells, clearly and rapidly fills the nanosensor device’s protective window formed by SU-8. Thus, it demonstrates that this SU-8 protective window does not prevent the blood-to-device free interaction, further allowing the analyte biomarkers detection and quantification. Additionally, Figure 3d shows an optical image of a 1 mm microneedle element after skin impalement into the forearm of a volunteer, exhibiting little visual residues along the entire length of the microneedle, indicating full penetration of the needle and reaching the required capillary depth.

Another possible location for *in vivo* analysis of protein biomarkers directly from capillary blood is illustrated in Figure 3e. In recent years, POC diagnostic devices research and development have tried to boost the use of capillary blood instead of venous blood since these tests would be more comfortable for patients and will provide a significant boost in quality-of-life and simplicity of the measurements. Finger pricking has been the method of choice for noncontinuous glucose measurements for diabetic individuals. While some research has been conducted on protein biomarkers detection from extracted whole capillary blood samples, these tests require manipulating the small volumes of extracted capillary blood and taking tens of minutes to hours to achieve results.^63,64^ However, these results show that PSA and many other protein biomarkers concentrations in capillary blood correlate well with their respective concentration in venous blood. Therefore, finger pricking can provide proper and straightforward means for direct *in vivo* diagnosis measurements when combined with methods for protein detection in nonmanipulated whole capillary blood specimens.^47^ Past investigations have shown that healthy individuals possess a capillary density higher than 60–200 capillaries per mm^2^.^65−69^ Thus, the transdermal penetration of our developed sensing microneedle elements, down to the required capillary depth, will lead to the rupture of capillary elements and the formation of a tiny capillary blood “pool” in the site of microneedle puncture. This formed capillary blood “pool” surrounding the sensing microneedle elements will lead to the surface capture of the protein biomarkers of interest to the electrical nanosensors devices and their subsequent quantitative detection.

**Figure 4:**
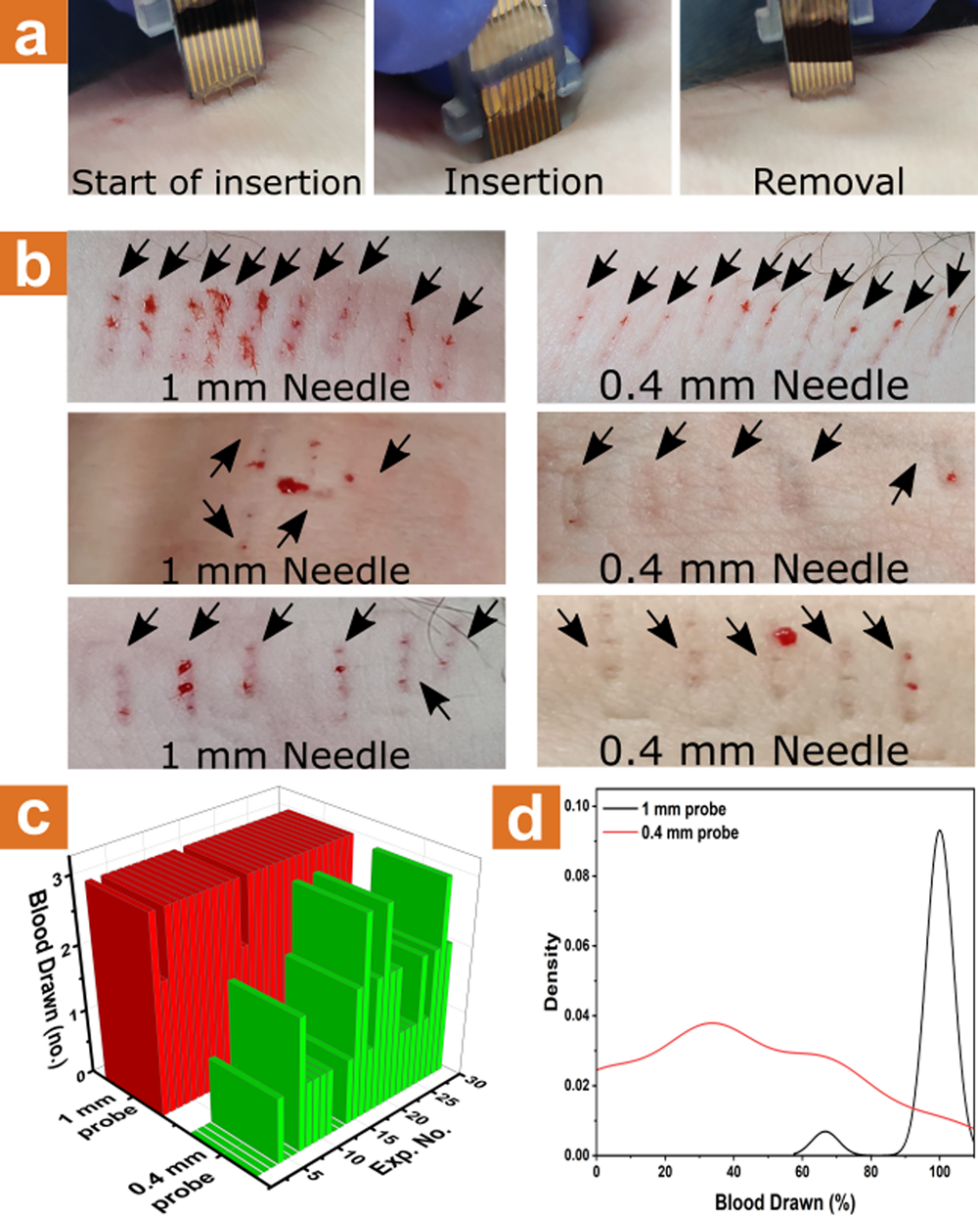
Skin insertion and contact with blood vessels. (a) Images of the insertion process of the microneedle array sensors into the forearm. (b) Images showing several insertion experiments of 1 mm microneedle array (left) and 0.4 mm microneedle array (right) to the forearm. (c) Summarized results of blood drawing from insertion experiments of 1 mm microneedle array (red) and 0.4 mm microneedle array (green) to the forearm. (d) Statistical distribution of blood drawing success percentage from 50 insertion experiments of 1 mm microneedle array (black) and 0.4 mm microneedle array (red) to the forearm.

Representative optical images of the microneedle insertion process through the skin in the forearm could be found in Figure 4a. The needles are shown to penetrate smoothly, with the entire microneedle array inserted in its total length. The chip’s handle is used as a self-limiting component for the entry depth and allows only the microneedle elements to be inserted into the dermal layer. Mechanical tests for quantifying the force needed for skin penetration were previously conducted using a pigs’ skin model,^24^ where different skin stiffness was mimicked by using a PDMS support for higher stiffness. Without the PDMS support, 1N force was required to penetrate the skin, and 0.2N was needed with higher stiffness. These results comply with previous microneedle skin penetration experiments.^24,70,71^ Notably, the needles did not break even after a 5N of applied force, thus proving mechanical robustness and safety.

Figure 4b displays results from three different healthy volunteers after applying 1 mm (left) and 400 μm (right) microneedle elements to penetrate their skin, while the resulting puncture sites were photographed postextraction. During insertion, minimal to no pain was reported by all volunteers. Notably, each penetration attempt using 1 mm microneedle elements resulted in the formation of a small drop of capillary blood for all three healthy volunteers tested, indicating that the 1 mm microneedle is sufficient for efficient protein biomarkers measuring purposes directly from capillary blood. Statistics of intradermal capillary blood “pool” formation experiments comparing the two needles lengths are shown in Figure 4c,d. A considerably higher success rate for the formation of capillary blood intradermal “pools” is achieved using the longer 1 mm microneedle chips, with almost 100% of all incidents leading to capillary blood pool formation.

Most of the SiNW-FET sensing devices in the published literature rely on the use of microfluidics for fluid exchange and sensing assays. To simplify *in vivo* sensing experiments, and due to the shape of our microneedles, no reliance on external microfluidics is required in our platform. Figure S5a depicts a representative bare nanodevice’s response to different pH media. The other pH solutions were made from 10 mM potassium phosphate monobasic and dibasic species to ensure identical ionic strength, thus eliminating the effects of different ionic strengths on the sensor’s signal. Each pH was measured twice by putting the microneedle sensor in the pH solution using a micromanipulator, removing the device, and reinserting it in order to see if any changes occurred. Once the device had reached the first plateau and stabilized, no further difference was seen between each insertion. All the SiNW elements in this work were coated with a two nm-thick alumina layer by atomic layer deposition. The coating allowed long and stable experiments to be conducted,^72^ although it is not a strict requirement for our current sensing experiments. The bare nanodevices exhibit a strong dependence on pH, as shown in Figure S5b. Additionally, the response variability of the nanodevices is negligible once the signal has stabilized, reaching up to 1% of the total averaged value (average over five cycles measured at the highest point).

**Figure 5:**
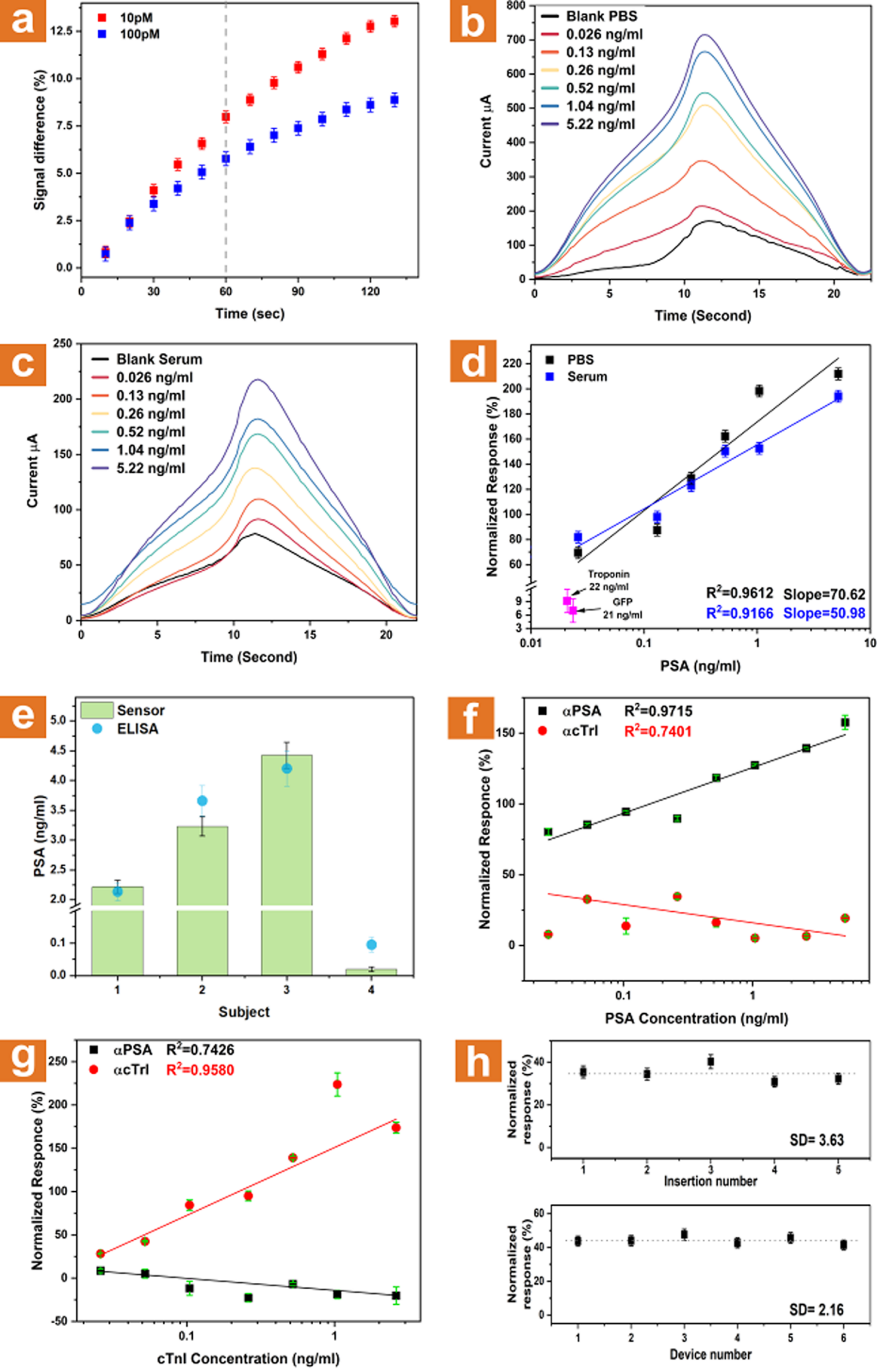
*In vivo* results of different measurements using the microneedle array. (a) Stabilization curves of PSA association at 10 pM (red curve) and 100 pM (blue curve) spiked-PBS solutions. The results indicate that the sensor requires approximately 60 s to achieve a differentiable signal. (b) One cycle close-up view taken once dissociation stabilization is achieved for PSA-spiked PBS buffer. (c) One cycle close-up view taken once dissociation stabilization is achieved for PSA-spiked serum. (d) Normalized response linear curves derived from (a) and (b). The dissociation phase was conducted in 5% EG in 100 μM phosphate buffer solution. Normalized reaction is in comparison to nonspiked buffer or serum, respectively. Pink data relates to nonspecific normalized response to 22 ng/mL cTnI and 21 ng/mL GFP. (e) *In vivo* intradermal capillary PSA concentrations were measured in four subjects using the microneedle array (green bars) compared to ELISA measurements of PSA concentration in venous blood (blue dots). (f) Multiplex experiment results of normalized response to PSA-spiked buffer from a device modified with PSA-specific antibody (αPSA, black curve) and a device modified with cTnI-specific antibody (αcTnI, red curve). (g) Multiplex experiment results of normalized response to cTnI-spiked buffer from a device modified with PSA-specific antibody (αPSA, black curve) and a device modified with cTnI-specific antibody (αcTnI, red curve). (i) Top: Deviation measurements performed on a single device via multiple entries to (100 pM spiked serum solution). Bottom: Variance measurements were performed between different devices via normalized response against a 100 pM spiked serum.

Prior to *in vivo* intradermal measurements in capillary blood, an *in vitro* test was conducted to see the device’s sensitivity toward the protein biomarker PSA. The test was separated into two regimes–specific association and dissociation. The association occurred at 1× phosphate buffer saline (PBS) solution spiked with different PSA concentrations. At the same time, the dissociation step was performed using a low ionic strength sensing buffer SB solution (0.01× of 10 mM phosphate buffer) with 5% added ethylene glycol (EG). Prior results using this method have shown the advantage of using EG as a dissociation inhibitor at a concentration of 50%.^47^Figure 5a shows the result of the *in vitro* experiments, where the PSA dissociation step was performed using a 1:1 SB:EG solution. The results demonstrate the fast plateau reached after several seconds of PSA association, where an apparent signal differentiation was achieved within 60 s. Therefore, the halted transdermal insertion of the microneedle array for only several tens of seconds would be sufficient for diagnostic purposes. Also, a 1:1 SB:EG ratio was chosen to be suitable for the dissociation period, giving slower dissociation rates of the specific biomarker molecules from the surface-confined antibody receptor units, all the while letting nonspecific bound moieties dissociate quicker from the sensing area. Therefore, the overall sensing measurement time is fast, with results that can be achieved in less than 3 min, significantly surpassing the time required for today’s biomarkers measurements via conventional blood tests.

Figure 5b depicts one cycle of an *in vitro* measurement as described above. The signal normalization was performed against the “clean” signal, measured in a 1× PBS solution without any spiked PSA. Similarly, Figure 5c represents one current cycle of *in vitro* measurements serum spiked with rising PSA concentrations. Signal normalization was performed against “clean” unspiked serum. The presented concentration measurements were normalized poststabilization in relation to the association regime plateau using the formation formula:
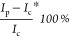
1where *I*_p_ is the current received from PSA-containing PBS solutions, and *I*_c_ is the current received from clean PSA-free PBS solution. Once calculated, the dissociation regimes were normalized using the resulting signal response percentages. The association regime plateau was used for the calibration since dealing with high ionic strength screens the electrical signals originating from protein association due to the short Debye screening length at these conditions. The results corresponding to each concentration were taken in relation to the stabilization of the “clean” solution’s signal.

A clear concentration-dependent sensing behavior is observed at increasing PSA concentrations, Figure 5d, which shows the linear curves of normalized response to different PSA concentrations in spiked PBS buffer (black curve) and in spiked serum (blue curve). The normalized response is taken as an average value measured after five consecutive cycles of dissociation regime. Specificity tests of the response of the microneedle-embedded SiNW array were conducted by measurements in the presence of high concentrations of nonspecific proteins. The anti-PSA modified array showed near-zero response to 22 ng/mL of cardiac troponin I (cTnI) and 21 ng/mL green fluorescent protein (GFP). Figure S6b compares one cycle of PSA measurement in the presence of 100-fold higher concentrations of GFP and cTnI. These results indicate the high specificity of our sensing microneedle devices for the specific detection of the desired biomarker antigens. Importantly, these results indicate low sensitivity to varying interferents, since measurements were performed in untreated serum.

Following *in vitro* calibration of the sensing devices, *in vivo* intradermal sensing measurements through finger pricking, through the 30−60 s-delayed insertion of the microneedle elements into the intradermal space (microneedle impalement is followed by a waiting period of 30−60 s before final removal followed by *ex-vivo* dissociation measurement) of human volunteers were performed, shown in Figure 5e (green columns); 4 subjects were tested: Subject 1 is a healthy 30-year-old male, Subject 2 is a healthy 50-year-old male, Subject 3 is a healthy 30-year-old male triathlete (cycling over 20 km weekly), and Subject 4 is a healthy 30-year-old female. The results show that subjects’ PSA levels are within the normal healthy range for male Subjects 1 and 2 and are slightly higher for male Subject 3, as was expected since long-distance daily bicycling routines are well-known to cause high PSA levels.^73,74^ Very low PSA levels were also expected for the female Subject 4 since normal PSA levels in healthy females are about 0–0.52 ng/mL.^75^ The PSA levels from *in vivo* microneedle sensing trials were further validated by performing a gold-standard PSA-specific enzyme-linked immunosorbent assay (ELISA) using venous blood from the same volunteers, Figure 5e (blue dots). ELISA results show that the *in vivo* capillary blood direct sensing performed by our microneedle arrays has the capability to measure the target PSA protein biomarker accurately and falls within the error range of the gold standard ELISA measurements. Additionally, the comodification of both total-PSA and free-PSA antibody receptors on different microneedle elements on a single sensing chip further could allow the ratiometric clinical assessment of the PSA^total^/PSA^free^ ratio. This ratio is clinically well-known to provide more sensitive and specific information for the diagnosis of prostate cancer.

The microneedle-embedded nanosensors array presented here poses multiple advantages for future POC medical diagnosis applications. First and foremost, the lack of need to invasively extract and manipulate blood samples by the direct intradermal capillary blood-based detection of proteins biomarkers provides a great leap in the field of medical diagnosis. This type of sensing platform can also be used in clinical situations where the amount of available blood is inherently small, such as in newborn infants, without the need to prick heels or fingers to extract blood samples for further *ex vivo* analysis. Second, the simple fabrication process allows redundancy in the number of active sensors, providing reliable results and a small margin of clinical errors.

A large number of functional microneedle elements also allows the multiplexed detection of various protein biomarkers in a single-prick. Each needle can be readily modified with a different antibody or bioreceptor, as illustrated in Figure 2a. Depending on the multiplexing level required, many antibodies can be modified upon a single microneedle element. This allows multiple biomarkers to be detected using a single-prick single-sensor microneedles-based platform. Results from a multiplexed experiment are shown in Figure 5f,g, where one needle out of a microneedle array has been modified with a PSA-specific antibody (αPSA) and the adjacent needle was modified with a cTnI-specific antibody (αcTnI). Both needles were exposed to varying concentrations of PSA and cTnI. The needle modified with αPSA showed a clear linear response to PSA, while the needle modified with αcTnI showed no meaningful response, Figure 5f, and when exposed to cTnI concentrations both the opposite response was exhibited by both needles, Figure 5g. An additional response curve to cTnI in blood could be found in Figure S9.

The response from the sensing microneedle array is shown to be highly accurate and reliable, Figure 5h, making it a potential candidate as a future POC diagnostic tool. Figure S10, which is derived from Figure 5h, shows the variation between each needle. Additionally, measurements of PSA-spiked serum and buffer by the same device produce near-identical curves, Figure S11. Therefore, the microneedles arrays are highly reliable and produce an extremely reproducible protein response. The difference between curves of different dies is expected to diminish with mass-production and process scale-up.

**Figure 6:**
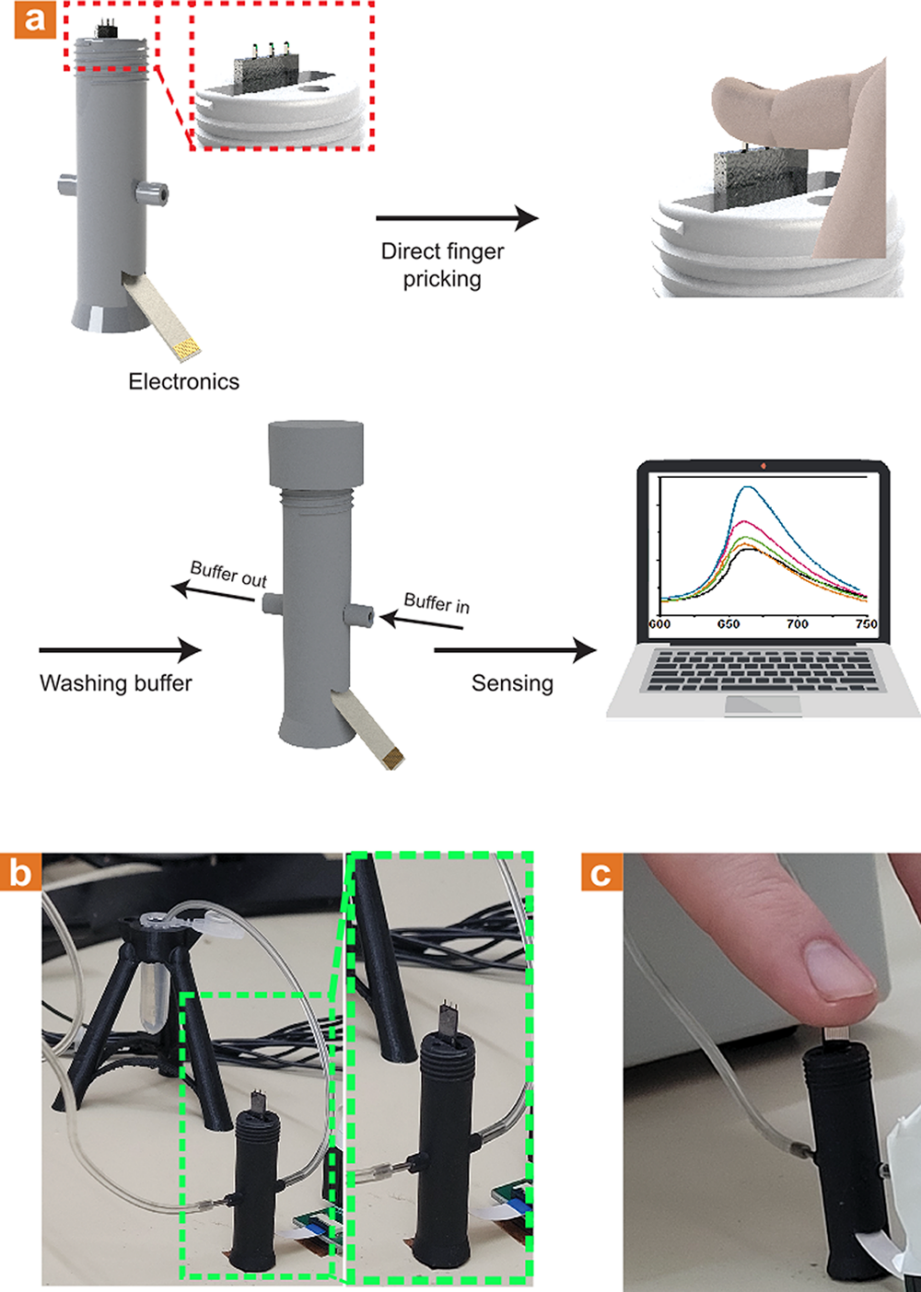
Laboratory-scale 3D-printed mount. (a) Illustration of the mount that enables the microneedle-based sensor operation. The mount chip is held for the user to prick his finger using the microneedles, followed by capping of the mount and consecutive washing in the appropriate buffer solution. (b) Optical images show the 3D-printed mount and the laboratory-scale system. (c) Optical image illustrating finger pricking using the 3D-printed mount.

Figure 6 depicts a laboratory-scale 3D-printed prototype for the real-world application of the microneedle-based sensing platform for the finger-prick blood extraction-free intradermal protein biomarkers detection. The 3D-printed device allows for the subject to safely and comfortably prick his own finger, followed by the performance of the rapid and straightforward ex-dermal dissociation-based detection and quantification of the protein biomarkers of interest.

The presented blood extraction-free microneedle sensing platform provides an enormous conceptual leap in the field of medical diagnosis in general and POC medical diagnosis in particular. These fields are currently dominated by time-consuming invasive and extensive high-volume blood extraction and manipulation steps, performed mainly by professional medical staff at centralized facilities. Our presented diagnosis paradigm, based on the application of microneedle-embedded chemically modified nanosensors array devices, allows the simultaneous intradermal penetration and *in-skin* capillary blood-based biomarkers quantitative sampling and detection. This ultimate POC platform combines prominently advantageous attributes such as minimal invasiveness, not requiring a blood sample, manipulation-free requirements for samples, clinically relevant high sensitivity and specificity, sensing accuracy, rapid detection turnover of under 3 min, and multiplexing capabilities for the detection of multiple protein biomarkers based on a single-prick single-chip direct approach.

## Conclusions

We have introduced a microneedle-embedded nano electrical sensors array platform for the intradermal, minimally invasive, and blood extraction-free platform for the clinical POC multiplexed detection of proteins biomarkers. This proposed diagnosis paradigm requires no extraction and ex-body manipulation procedures of large-volume venous blood samples, which is currently ordinary in all diagnostic tests. Our miniature on-chip platform allows the direct intradermal probing of the prick-triggered capillary blood ’pool’ formed by the microneedle in the puncture site, only a few hundreds of nanoliters in volume, and the concomitant *in-skin* quantitative capturing of the protein biomarkers of interest, followed by the microneedle removal and *ex vivo* biomarkers levels quantification. The microneedle length required to effectively and smoothly breach the skin layers down to the required capillary depth was experimentally determined as ≥1 mm, with a nearly 100% success rate of reaching blood capillaries’ required depth after each insertion event. Also, an approach for the physical protection of the molecular recognition layer was developed by creating a SU-8-based open window structure. This micrometers-high SU-8 protecting window allows the microneedles’ skin penetration without the “wiping off” effect of the antibody recognition layer on the active sensing area. Still, this open window lets the fast and complete wetting of the sensing area when surrounded by the pricking-triggered capillary blood “pool”, thus allowing the free *in-skin* interaction of the embedded nanodevices sensing array with the surrounding blood sample. The microneedles’ array has shown a detection sensitivity in the sub-pM range and has been preliminary applied clinically for the intradermal direct *in vivo* blood extraction-free detection of PSA on healthy human volunteers. These detection results directly correlate with values measured from venous blood extracted samples by gold-standard ELISA analysis. Furthermore, the top-down process presented in this work, based on common fabrication techniques, allows multiple vital advantages, such as high device redundancy for reliable results, ease of integration with future drug delivery applications, scalable and cost-effective process, and, most importantly, multiplex detection of multiple biomarkers in a single-prick single-chip device. We believe our absolute POC diagnostics paradigm method could in the future eliminate the need for currently well-established venous blood extraction and sample manipulation-based clinical approaches for disease diagnosis.

## Methods

### Materials and Chemicals

For this investigation, the following materials and chemicals were obtained: an 8-in. SOI wafer (Silicon Valley Microelectronics), acetone (9005-68, J.T. Baker), isopropanol (9079-05, J.T. Baker), deionized water (18 MΩ·cm), phosphate buffer (PB, 10 mM, pH 8.5), phosphate buffer (SB, 155 μM, ∼ pH 8.0), phosphate buffer saline (PBS, 10 mM, pH 7.4, with 2.7 mM KCl and 137 mM NaCl), glutaraldehyde solution (50 wt % in H_2_O, G7651, Sigma-Aldrich), (3-aminopropyl)-dimethyl-ethoxysilane (APDMES, SIA0603.0–5g, Gelest), human PSA protein (ABCAM, ab78528) PSA antibody (ABCAM, ab75684), cardiac troponin I protein (cTnI, ABCAM, ab207624), cardiac troponin I antibody (ABCAM, ab38210), GFP protein (ABCAM, ab84191), GFP antibody (ABCAM, ab1218) Alexa-488 NHS (A20000, Thermo Fisher), PDMS (Sylgard), LOR5A (Kayaku Advanced Materials), LOR7A (Kayaku Advanced Materials), LOR10A (Kayaku Advanced Materials), SU8 2000.5 (Kayaku Advanced Materials), SU8 3005 (Kayaku Advanced Materials), AZ1505 (MicroChemicals), AZ4562 (MicroChemicals), PMGI SF15 (Kayaku Advanced Materials), buffered oxide etchant 6:1 (BOE, Transene), gold etchant TFE (Transene), chromium cermet etchant (Transene), *N*-methyl-2-pyrrolidone (NMP, J.T. Baker), hydrogen peroxide (30% in water, Bio-Lab), sulfuric acid (95–98%, Bio-Lab), methyl methacrylate (MMA, EL6, Kayaku Advanced Materials), poly(methyl methacrylate) (PMMA, A4, Kayaku Advanced Materials), AZ726 (MicroChemicals), methyl isobutyl ketone (MIBK 1:3, Kayaku Advanced Materials), hydrofluoric acid (48%, Sigma-Aldrich), tetramethylammonium hydroxide (10% in water, Sigma-Aldrich), AZ400 K (MicroChemicals).

### Nanowire Fabrication

First, 30 mm × 30 mm SOI dies were thoroughly cleaned using acetone, IPA, and DIW and were dipped in a 1:3 H_2_O_2_:H_2_SO_4_ piranha solution for 2 min. Following a 2 min 60W O_2_ plasma, the dies were dipped in a 6:1 BOE to remove the native oxide and were thoroughly washed with DIW. LOR5A and AZ1505 were spin-coated on the dies using 500 rpm for 5 s, followed by 4000 rpm for 45 s. The dies were baked at 180 °C for 5 min following the LOR5A spin coat and were baked at 100 °C for 1 min following the AZ1505 spin coat process. E-beam markers were exposed using UV lithography and were developed for 1 min in AZ726 (MicroChemicals), followed by a thorough wash in DIW. The markers were evaporated with 5 nm Cr and 30 nm Au and put in NMP to resist lift-off.

Once done, the dies were coated with MMA EL6 and PMMA A4, using 500 rpm for 3 s and 5000 rpm for 60 s. The die was baked at 180 °C for 3 and 1 min, respectively. E-beam lithography (Raith 150) with 10 kV was used to expose the PMMA layer, with the wires exposed using 10 μm aperture and 140 μC/cm^2^ and the pads exposed using 60 μm aperture and 120 μC/cm^2^. Development took place using 1:3 MIBK (methyl isobutyl ketone): IPA solution for 1 min, followed by a thorough rinse in IPA. The exposed wire pattern was evaporated with 5 nm Cr and 30 nm Au and was placed in acetone for lift-off.

The dies were cleaned in IPA and DIW and were put in 60W O_2_ plasma for 2 min. The native oxide was removed using a 1:9 diluted 48% HF solution for 10 s and was directly placed, without rinsing, in a 10% TMAH solution heated to 75 °C. After approximately 30 s, the die changed its color, indicating the dissolution of the device layer, and was rinsed in DIW. The Au and Cr were removed using appropriate etchants for 2 min each while thoroughly washing in DIW in between etchants.

### Electrodes’ Fabrication

The dies were spin-coated with LOR5A and AZ1505 as described above. Outer pads were exposed and developed in AZ726 for 1 min, followed by a thorough rinse in DIW. The outer pads were evaporated with 5 nm Cr and 60 nm Au and were placed in warm NMP for lift-off. The dies were then washed in acetone, IPA, and DIW and were placed in an ozone generator for 3 min. LOR7A and AZ1505 were spin-coated as described above. Inner pads were exposed and developed as the outer pads. The inner pads were evaporated using 10 nm Ti, 90 nm Pd, and 5 nm Ti and were placed for the passivation process prior to lift off. The passivation took place via approximately 80 nm SiO_2_ deposition using 200W ICP, 30W Bias, 95 mTorr, 80 °C, 140 sccm N_2_O, and 14 sccm 2% SiH_4_/Ar for 20 min in a plasma-enhanced chemical vapor deposition (PECVD, Axis Benchmark 800 ICP) system. The dies were then placed in warm NMP for lift-off. Once the process was done, rapid thermal processing (RTP, AnnealSys) was used to create ohmic contacts between the Ti/Si interface. The dies were heated to 450 °C in 5 s and remained for an extra 20 s in a forming gas environment (2% H_2_ in N_2_).

### Crevice Fabrication and Thinning of Needles

In order to create the opening for the sensing area, SU8 was used. SU8 2000.5 was spin-coated using 300 rpm for 5 s, following 3000 rpm for 30 s. The die was baked for 5 min at 95 °C. After UV exposure, the dies were baked for 1 min at 95 °C. The dies were developed in a designated SU8 developer for 1 min, followed by an IPA rinse. Consecutively, SU8 3005 was coated using the same program as above. The die was baked for 1 min at 65 °C, following 10 min at 95 °C. After exposure, the dies were baked for 4 min at 95 °C and developed as above.

LOR10A was dispensed to protect the nanowire region from the dicing operation, as the dicing is done on the backside. The dies were thinned using automatic dicer (Disco DAD 3350) via lowering the saw up to a depth that leaves approximately 250 μm thickness (around 500 μm) and moving laterally in steps smaller than the total width of the saw (e.g., if the saw was 200 μm thick, the steps were set to 90 μm). Once done, LOR10A was removed in NMP.

### Formation of Needles

Prior to the deep silicon etching step, the BOX layer was removed. PMGI SF-15 was spin-coated at 500 rpm for 5 s and 1500 rpm for 45 s and was baked at 180 °C for 5 min. AZ4562 photoresist was spin-coated using the same parameters and baked at 115 °C for 1.5 min. The etch mask was exposed five consecutive times with 25 s stops in between. The dies were placed in DIW for 4 min following the exposure and developed in 1:2.5 diluted AZ400 K developer for 4 min. The oxide layer was removed using reactive ion etching (RIE, Oerlikon) using 200W forward bias, 40 sccm CF_4_, 5 sccm O_2_, and 6 sccm Ar for 23 min at room temperature. The complete removal of the oxide was determined via an interferometer. The resists were removed in NMP.

In order to protect the fabricated SiNW from possible ion damage, the thick resist was applied before deep reactive ion etching (DRIE, Deep RIE Versaline DSE). PMGI SF-15 was spin-coated, as discussed above. Two layers of AZ4562 were spin-coated using the same spin parameters as above. The first layer was baked at 90 °C for 3 min, and the second layer was baked at 115 °C for 2.5 min. Once the etch mask was exposed, as discussed above, the dies were placed in DIW for 5 min and were developed in 1:2.5 diluted AZ400 K developer for 8 min. After development, the dies were subjected to flood exposure of 400 mJ/cm^2^. The dies were placed in the DRIE using heatsink grease (Dow Corning 340 Heat Sink Compound Grease) and were etched using a 3-step process for 300 loops. Once done, the dies were diced and separated into individual microneedle array sensors, and the remaining resists were removed in warm NMP.

### Antibody Modification

Prior to the modification process, the microneedle array sensor was mounted on a 3D-printed holder (Form3 printer, Fromlabs) and was wire bonded to a flexible PCB. The mounted sensor was then placed in the ozone generator for 7 min to generate silanol groups on the SiNWs surface. The mounted array was placed in 200 μL of 95% APDMES solution for 1 h in an Ar-filled glovebox. The sensor was then placed in 150 μL of toluene to wash the remaining APDMES solution and was thoroughly rinsed with IPA and placed at 100 °C for 30 min to evaporate the remaining solvents completely.

Phosphate buffer (PB) was prepared by mixing 10 mM potassium phosphate monobasic solution and 10 mM potassium phosphate dibasic solution to pH 8.5. Then 1 ml of a 50% Glutaraldehyde solution (Sigma-Aldrich) was diluted in 5 mL of prepared PB with 50 mg of added NaCNBH_3_. The microneedle array sensor was dipped in 200 μL of the above solution for 1 h and was consecutively rinsed with DIW, IPA, and DIW again.

Antihuman PSA in 0.030 mg/mL concentration was used for the modification. The antibody was first centrifuged in a desalting column to clean and purify the protein properly. The anti-PSA was diluted to 30 μg/mL for the modification using a prepared solution of 5 mL of PB and 50 mg of NaCNBH_3_. The microneedle array was dipped in 200 μL of the antibody solution and was placed at 4 °C overnight.

Blocking solution was prepared using 150 μL of ethanolamine in 20 mL PB with 50 mg NaCNBH_3_, which was titrated back to pH 8.5 using HCl. Then 200 μL of the above solution was used for 2 h to block all unreacted aldehyde groups. The microneedle array was then thoroughly washed in PB by placing the sensor in a 200 μL solution of clean PB for 10 min. This process was repeated a total of three times before sensing experiments.

Anti-GFP and anti-cTnI were modified using the same method and the same concentrations to verify the viability of the modification properly.

### Array Cleaning Prior to Skin Insertion

The microneedle arrays were washed well in autoclaved PB; the insertion area was sterilized by rubbing ethanol.

### *In Vitro* and *In Vivo* Electrical Measurements

Electrical measurements were performed by varying the gate voltage in order to choose the gate voltage in which the most significant change in current was measured as a factor of concentration changes. The gate voltage was varied between −0.7 V and 0.3 V, and the source-drain voltage was constantly applied using 0.2 V. The *in vitro* measurements took place either in 1× phosphate buffer saline (PBS) or as-received bovine serum. The microneedle array sensor was placed inside an Eppendorf containing a 2 mL solution of either unspiked (“clean”) or PSA-spiked solutions in different concentrations until stabilization (approximately 8 min). The desorption took place in a 5% EG solution in sensing buffer (phosphate buffer diluted by a factor of 100) using 2 mL solutions as well, until stabilization (approximately 10 min). *In vivo* measurements in capillary blood were performed by full penetration of the microneedle array into the volunteer’s skin (arm or fingertip), with the microneedle probing allowed to occur for 1 min before final microneedle removal, followed by the final quantitative sensing analysis.

### Material Characterization

XPS measurements were performed using the 5600 Multi-Technique System (PHI, U.S.A.). SEM images were taken using Environmental SEM (Quanta 200FEG, Jeol Co.).

### ELISA Measurements

ELISA kit to quantify total PSA was purchased from ABCAM (ab113327). The measurement protocol is as follows:

A 96-well plate coated with an antibody specific for Human PSA was used. Next, 100 μL of standard solutions and samples (see elaborated below) were pipetted into the wells. The wells were washed thoroughly, and a 100 μL of biotinylated secondary antibody to human PSA was added. The wells were washed thoroughly again; then 100 μL of HRP-conjugated streptavidin was added to each well. The wells were again washed, and 100 μL of TMB substrate solution was added, developing a blue color in proportion to the amount of PSA bound. Adding 50 μL of Stop Solution changes the color from blue to yellow, and the intensity of the color is measured at 450 nm.

Approximately 5 mL of venous blood was extracted and centrifuged to coagulate and remove the red blood cells. The test was performed directly on the separated plasma fluid remaining after 2-fold dilution in Assay Diluent B provided in the kit.

A standard PSA solution of 50 000 pg/mL was diluted in Assay Diluent B to perform calibration curve measurements of 10.24–2500 pg/mL (see Figure S8).

## Supporting Information Available

SEM images of the microneedle-based FET, XPS measurements spectra, EIS of the modification steps, fluorescence microscopy images of SU-8 window, electrical pH measurements, enzyme-linked immunosorbent assay calibration curve for PSA, and current data corresponding to curves in Figure 5 (PDF)

## References

[ref1] Yu H.; Lu Y.; Zhou Y.; Wang F.; He F.; Xia X. A Simple, Disposable Microfluidic Device for Rapid Protein Concentration and Purificationvia Direct-Printing. Lab Chip 2008, 8, 1496–1501. 10.1039/b802778a. 18818804

[ref2] Kim P.; Kim S. J.; Han J.; Suh K. Y. Stabilization of Ion Concentration Polarization Using a Heterogeneous Nanoporous Junction. Nano Lett. 2010, 10, 16–23. 10.1021/nl9023319. 20017532 PMC2806642

[ref3] Dhopeshwarkar R.; Crooks R. M.; Hlushkou D.; Tallarek U. Transient Effects on Microchannel Electrokinetic Filtering with an Ion-Permselective Membrane. Anal. Chem. 2008, 80, 1039–1048. 10.1021/ac7019927. 18197694

[ref4] Jeun M.; Lee H. J.; Park S.; Do E.; Choi J.; Sung Y.-N.; Hong S.-M.; Kim S.-Y.; Kim D.-H.; Kang J. Y.; Son H.-N.; Joo J.; Song E. M.; Hwang S. W.; Park S. H.; Yang D.-H.; Ye B. D.; Byeon J.-S.; Choe J.; Yang S.-K.; et al. A Novel Blood-Based Colorectal Cancer Diagnostic Technology Using Electrical Detection of Colon Cancer Secreted Protein-2. Adv. Sci. 2019, 6, 1802115 10.1002/advs.201802115. PMC6548955 31179210

[ref5] Gao X.; Boryczka J.; Kasani S.; Wu N. Enabling Direct Protein Detection in a Drop of Whole Blood with an “On-Strip” Plasma Separation Unit in a Paper-Based Lateral Flow Strip. Anal. Chem. 2021, 93, 1326–1332. 10.1021/acs.analchem.0c02555. 33347264

[ref6] Biagini R. E.; Sammons D. L.; Smith J. P.; MacKenzie B. A.; Striley C. A. F.; Snawder J. E.; Robertson S. A.; Quinn C. P. Rapid, Sensitive, and Specific Lateral-Flow Immunochromatographic Device to Measure Anti-Anthrax Protective Antigen Immunoglobulin G in Serum and Whole Blood. Clin. Vaccine Immunol. 2006, 13, 541–546. 10.1128/CVI.13.5.541-546.2006. 16682473 PMC1459649

[ref7] Espy R. D.; Manicke N. E.; Ouyang Z.; Cooks R. G. Rapid Analysis of Whole Blood by Paper Spray Mass Spectrometry for Point-of-Care Therapeutic Drug Monitoring. Analyst 2012, 137, 2344–2349. 10.1039/c2an35082c. 22479698

[ref8] McCaughey E. J.; Vecellio E.; Lake R.; Li L.; Burnett L.; Chesher D.; Braye S.; Mackay M.; Gay S.; Badrick T. C.; Westbrook J. I.; Georgiou A. Current Methods of Haemolysis Detection and Reporting as a Source of Risk to Patient Safety: A Narrative Review. Clin. Biochem. Rev. 2016, 37, 143. 28167844 PMC5242478

[ref9] Heireman L.; Van Geel P.; Musger L.; Heylen E.; Uyttenbroeck W.; Mahieu B. Causes, Consequences and Management of Sample Hemolysis in the Clinical Laboratory. Clin. Biochem. 2017, 50, 1317–1322. 10.1016/j.clinbiochem.2017.09.013. 28947321

[ref10] Wu C. C.; Lin H. Y.; Wang C. P.; Lu L. F.; Yu T. H.; Hung W. C.; Houng J. Y.; Chung F. M.; Lee Y. J.; Hu J. J. Evaluation of a Rapid Quantitative Determination Method of PSA Concentration with Gold Immunochromatographic Strips. BMC Urol 2015, 15, 109 10.1186/s12894-015-0105-7. 26530738 PMC4630854

[ref11] Kemper D. W.; Semjonow V.; de Theije F.; Keizer D.; van Lippen L.; Mair J.; Wille B.; Christ M.; Geier F.; Hausfater P.; Pariente D.; Scharnhorst V.; Curvers J.; Nieuwenhuis J. Analytical Evaluation of a New Point of Care System for Measuring Cardiac Troponin I. Clin. Biochem. 2017, 50, 174–180. 10.1016/j.clinbiochem.2016.11.011. 27847339

[ref12] Lippi G.; Plebani M.; Di Somma S.; Cervellin G. Hemolyzed Specimens: A Major Challenge for Emergency Departments and Clinical Laboratories. Crit. Rev. Clin. Lab. Sci. 2011, 48, 143–153. 10.3109/10408363.2011.600228. 21875312

[ref13] Sawant R. B.; Jathar S. K.; Rajadhyaksha S. B.; Kadam P. T. Red Cell Hemolysis during Processing and Storage. Asian J. Transfus. Sci. 2007, 1, 47 10.4103/0973-6247.33446. 21938232 PMC3168119

[ref14] Sharma S.; Huang Z.; Rogers M.; Boutelle M.; Cass A. E. G. Evaluation of a Minimally Invasive Glucose Biosensor for Continuous Tissue Monitoring. Anal. Bioanal. Chem. 2016 40829 2016, 408, 8427–8435. 10.1007/s00216-016-9961-6. PMC5116314 27744480

[ref15] Bal S. M.; Caussin J.; Pavel S.; Bouwstra J. A. In Vivo Assessment of Safety of Microneedle Arrays in Human Skin. Eur. J. Pharm. Sci. 2008, 35 (3), 193–202. 10.1016/j.ejps.2008.06.016. 18657610

[ref16] Noh Y. W.; Kim T. H.; Baek J. S.; Park H. H.; Lee S. S.; Han M.; Shin S. C.; Cho C. W. In Vitro Characterization of the Invasiveness of Polymer Microneedle against Skin. Int. J. Pharm. 2010, 397, 201–205. 10.1016/j.ijpharm.2010.06.050. 20619328

[ref17] El-Laboudi A.; Oliver N. S.; Cass A.; Johnston D. Use of Microneedle Array Devices for Continuous Glucose Monitoring: A Review. Diabetes Technol. Ther. 2013, 15, 101–115. 10.1089/dia.2012.0188. 23234256

[ref18] Yu J.; Wang J.; Zhang Y.; Chen G.; Mao W.; Ye Y.; Kahkoska A. R.; Buse J. B.; Langer R.; Gu Z. Glucose-Responsive Insulin Patch for the Regulation of Blood Glucose in Mice and Minipigs. Nat. Biomed. Eng. 2020, 4, 499–506. 10.1038/s41551-019-0508-y. 32015407 PMC7231631

[ref19] Yu J.; Zhang Y.; Ye Y.; DiSanto R.; Sun W.; Ranson D.; Ligler F. S.; Buse J. B.; Gu Z. Microneedle-Array Patches Loaded with Hypoxia-Sensitive Vesicles Provide Fast Glucose-Responsive Insulin Delivery. Proc. Natl. Acad. Sci. U. S. A. 2015, 112, 8260–8265. 10.1073/pnas.1505405112. 26100900 PMC4500284

[ref20] Yoon Y.; Lee G. S.; Yoo K.; Lee J.-B. Fabrication of a Microneedle/CNT Hierarchical Micro/Nano Surface Electrochemical Sensor and Its In-Vitro Glucose Sensing Characterization. Sensors 2013, 13, 16672–16681. 10.3390/s131216672. 24304643 PMC3892836

[ref21] Wang P. M.; Cornwell M.; Prausnitz M. R. Minimally Invasive Extraction of Dermal Interstitial Fluid for Glucose Monitoring Using Microneedles. Diabetes Technol. Ther. 2005, 7, 131 10.1089/dia.2005.7.131. 15738711

[ref22] Miller P. R.; Taylor R. M.; Tran B. Q.; Boyd G.; Glaros T.; Chavez V. H.; Krishnakumar R.; Sinha A.; Poorey K.; Williams K. P.; Branda S. S.; Baca J. T.; Polsky R. Extraction and Biomolecular Analysis of Dermal Interstitial Fluid Collected with Hollow Microneedles. Commun. Biol. 2018, 1, 173 10.1038/s42003-018-0170-z. 30374463 PMC6197253

[ref23] Teymourian H.; Tehrani F.; Mahato K.; Wang J. Lab under the Skin: Microneedle Based Wearable Devices. Adv. Healthc. Mater. 2021, 10, 2002255 10.1002/adhm.202002255. 33646612

[ref24] Heifler O.; Borberg E.; Harpak N.; Zverzhinetsky M.; Krivitsky V.; Gabriel I.; Fourman V.; Sherman D.; Patolsky F. Clinic-on-a-Needle Array toward Future Minimally Invasive Wearable Artificial Pancreas Applications. ACS Nano 2021, 15, 12019–12033. 10.1021/acsnano.1c03310. PMC8397432 34157222

[ref25] Mishra R. K.; Vinu Mohan A. M.; Soto F.; Chrostowski R.; Wang J. A Microneedle Biosensor for Minimally-Invasive Transdermal Detection of Nerve Agents. Analyst 2017, 142, 918–924. 10.1039/C6AN02625G. 28220163

[ref26] Corrie S. R.; Fernando G. J. P.; Crichton M. L.; Brunck M. E. G.; Anderson C. D.; Kendall M. A. F. Surface-Modified Microprojection Arrays for Intradermal Biomarker Capture, with Low Non-Specific Protein Binding. Lab Chip 2010, 10, 2655–2658. 10.1039/c0lc00068j. 20820632

[ref27] Sharma S.; El-Laboudi A.; Reddy M.; Jugnee N.; Sivasubramaniyam S.; El Sharkawy M.; Georgiou P.; Johnston D.; Oliver N.; Cass A. E. G. A Pilot Study in Humans of Microneedle Sensor Arrays for Continuous Glucose Monitoring. Anal. Methods 2018, 10, 2088–2095. 10.1039/C8AY00264A.

[ref28] Romanyuk A. V.; Zvezdin V. N.; Samant P.; Grenader M. I.; Zemlyanova M.; Prausnitz M. R. Collection of Analytes from Microneedle Patches. Anal. Chem. 2014, 86, 10520–10523. 10.1021/ac503823p. 25367229 PMC4222632

[ref29] Dinesh B.; Saraswathi R. Electrochemical Synthesis of Nanostructured Copper-Curcumin Complex and Its Electrocatalytic Application towards Reduction of 4-Nitrophenol. Sensors Actuators B Chem. 2017, 253, 502–512. 10.1016/j.snb.2017.06.149.

[ref30] Ishai M. B.; Patolsky F. Shape-and Dimension-Controlled Single-Crystalline Silicon and Sige Nanotubes: Toward Nanofluidic Fet Devices. J. Am. Chem. Soc. 2009, 131, 3679–3689. 10.1021/ja808483t. 19226180

[ref31] Ben-Ishai M.; Patolsky F. A Route to High-Quality Crystalline Coaxial Core/Multishell Ge@Si(GeSi)n and Si@(GeSi)n Nanowire Heterostructures. Adv. Mater. 2010, 22, 902–906. 10.1002/adma.200902815. 20217814

[ref32] Pevzner A.; Engel Y.; Elnathan R.; Tsukernik A.; Barkay Z.; Patolsky F. Confinement-Guided Shaping of Semiconductor Nanowires and Nanoribbons: “Writing with Nanowires.. Nano Lett. 2012, 12, 7–12. 10.1021/nl201527h. 22142384

[ref33] Weizmann Y.; Patolsky F.; Popov I.; Willner I. Telomerase-Generated Templates for the Growing of Metal Nanowires. Nano Lett. 2004, 4, 787–792. 10.1021/nl049939z.

[ref34] Jiang X.; Xiong Q.; Nam S.; Qian F.; Li Y.; Lieber C. M. InAs/InP Radial Nanowire Heterostructures as High Electron Mobility Devices. Nano Lett. 2007, 7, 3214–3218. 10.1021/nl072024a. 17867718

[ref35] Harpak N.; Davidi G.; Granot E.; Patolsky F. Diversely Doped Uniform Silicon Nanotube Axial Heterostructures Enabled by “Dopant Reflection.. Langmuir 2021, 37, 1247–1254. 10.1021/acs.langmuir.0c03249. 33417463

[ref36] Kuhn S.; Asenbaum P.; Kosloff A.; Sclafani M.; Stickler B. A.; Nimmrichter S.; Hornberger K.; Cheshnovsky O.; Patolsky F.; Arndt M. Cavity-Assisted Manipulation of Freely Rotating Silicon Nanorods in High Vacuum. Nano Lett. 2015, 15, 5604–5608. 10.1021/acs.nanolett.5b02302. 26167662 PMC4538454

[ref37] Yeor-Davidi E.; Zverzhinetsky M.; Krivitsky V.; Patolsky F. Real-Time Monitoring of Bacterial Biofilms Metabolic Activity by a Redox-Reactive Nanosensors Array. J. Nanobiotechnology 2020, 18, 81 10.1186/s12951-020-00637-y. 32448291 PMC7247256

[ref38] Borberg E.; Pashko S.; Koren V.; Burstein L.; Patolsky F. Depletion of Highly Abundant Protein Species from Biosamples by the Use of a Branched Silicon Nanopillar On-Chip Platform. Anal. Chem. 2021, 93, 14527–14536. 10.1021/acs.analchem.1c03506. 34668374 PMC8592501

[ref39] Harpak N.; Davidi G.; Schneier D.; Menkin S.; Mados E.; Golodnitsky D.; Peled E.; Patolsky F. Large-Scale Self-Catalyzed Spongelike Silicon Nano-Network-Based 3D Anodes for High-Capacity Lithium-Ion Batteries. Nano Lett. 2019, 19, 1944–1954. 10.1021/acs.nanolett.8b05127. 30742440

[ref40] Harpak N.; Davidi G.; Melamed Y.; Cohen A.; Patolsky F. Self-Catalyzed Vertically Aligned Carbon Nanotube-Silicon Core-Shell Array for Highly Stable, High-Capacity Lithium-Ion Batteries. Langmuir 2020, 36, 889–896. 10.1021/acs.langmuir.9b03424. 31948231

[ref41] Harpak N.; Davidi G.; Cohen A.; Raz A.; Patolsky F. Thermally-Treated Nanowire-Structured Stainless-Steel as an Attractive Cathode Material for Lithium-Ion Batteries. Nano Energy 2020, 76, 105054 10.1016/j.nanoen.2020.105054.

[ref42] Patolsky F.; Zheng G.; Hayden O.; Lakadamyali M.; Zhuang X.; Lieber C. M. Electrical Detection of Single Viruses. Proc. Natl. Acad. Sci. U. S. A. 2004, 101, 14017–14022. 10.1073/pnas.0406159101. 15365183 PMC521090

[ref43] Zheng G.; Patolsky F.; Cui Y.; Wang W. U.; Lieber C. M. Multiplexed Electrical Detection of Cancer Markers with Nanowire Sensor Arrays. Nat. Biotechnol. 2005, 23, 1294–1301. 10.1038/nbt1138. 16170313

[ref44] Krivitsky V.; Zverzhinetsky M.; Krivitsky A.; Hsiung L.-C.; Naddaka V.; Gabriel I.; Lefler S.; Conroy J.; Burstein L.; Patolsky F. Cellular Metabolomics by a Universal Redox-Reactive Nanosensors Array: From the Cell Level to Tumor-on-a-Chip Analysis. Nano Lett. 2019, 19, 2478–2488. 10.1021/acs.nanolett.9b00052. 30884235

[ref45] Lichtenstein A.; Havivi E.; Shacham R.; Hahamy E.; Leibovich R.; Pevzner A.; Krivitsky V.; Davivi G.; Presman I.; Elnathan R.; Engel Y.; Flaxer E.; Patolsky F. Supersensitive Fingerprinting of Explosives by Chemically Modified Nanosensors Arrays. Nat. Commun. 2014, 5, 4195 10.1038/ncomms5195. 24960270

[ref46] Meir R.; Zverzhinetsky M.; Harpak N.; Borberg E.; Burstein L.; Zeiri O.; Krivitsky V.; Patolsky F. Direct Detection of Uranyl in Urine by Dissociation from Aptamer-Modified Nanosensor Arrays. Anal. Chem. 2020, 92, 12528–12537. 10.1021/acs.analchem.0c02387. 32842739

[ref47] Zverzhinetsky M.; Krivitsky V.; Patolsky F. Direct Whole Blood Analysis by the Antigen-Antibody Chemically-Delayed Dissociation from Nanosensors Arrays. Biosens. Bioelectron. 2020, 170, 112658 10.1016/j.bios.2020.112658. 33035904

[ref48] Krivitsky V.; Zverzhinetsky M.; Patolsky F. Antigen-Dissociation from Antibody-Modified Nanotransistor Sensor Arrays as a Direct Biomarker Detection Method in Unprocessed Biosamples. Nano Lett. 2016, 16, 6272–6281. 10.1021/acs.nanolett.6b02584. 27579528

[ref49] Borberg E.; Zverzhinetsky M.; Krivitsky A.; Kosloff A.; Heifler O.; Degabli G.; Soroka H. P.; Fainaro R. S.; Burstein L.; Reuveni S.; Diamant H.; Krivitsky V.; Patolsky F. Light-Controlled Selective Collection-and-Release of Biomolecules by an On-Chip Nanostructured Device. Nano Lett. 2019, 19, 5868–5878. 10.1021/acs.nanolett.9b01323. 31381354

[ref50] Krivitsky V.; Hsiung L. C.; Lichtenstein A.; Brudnik B.; Kantaev R.; Elnathan R.; Pevzner A.; Khatchtourints A.; Patolsky F. Si Nanowires Forest-Based on-Chip Biomolecular Filtering, Separation and Preconcentration Devices: Nanowires Do It All. Nano Lett. 2012, 12, 4748–4756. 10.1021/nl3021889. 22852557

[ref51] Yang X.; Gao A.; Wang Y.; Li T. Wafer-Level and Highly Controllable Fabricated Silicon Nanowire Transistor Arrays on (111) Silicon-on-Insulator (SOI) Wafers for Highly Sensitive Detection in Liquid and Gaseous Environments. Nano Res. 2018, 11, 1520–1529. 10.1007/s12274-017-1768-z.

[ref52] Li D.; Chen H.; Fan K.; Labunov V.; Lazarouk S.; Yue X.; Liu C.; Yang X.; Dong L.; Wang G. A Supersensitive Silicon Nanowire Array Biosensor for Quantitating Tumor Marker CtDNA. Biosens. Bioelectron. 2021, 181, 113147 10.1016/j.bios.2021.113147. 33773219

[ref53] Malsagova K. A.; Ivanov Y. D.; Pleshakova T. O.; Kaysheva A. L.; Shumov I. D.; Kozlov A. F.; Archakov A. I.; Popov V. P.; Fomin B. I.; Latyshev A. V. A SOI-Nanowire Biosensor for the Multiple Detection of D-NFATc1 Protein in the Serum. Anal. Methods 2015, 7, 8078–8085. 10.1039/C5AY01866H.

[ref54] Stern E.; Vacic A.; Reed M. A. Semiconducting Nanowire Field-Effect Transistor Biomolecular Sensors. IEEE Trans. Electron Devices 2008, 55, 3119–3130. 10.1109/TED.2008.2005168.

[ref55] Beckman R. A.; Johnston-Halperin E.; Melosh N. A.; Luo Y.; Green J. E.; Heath J. R. Fabrication of Conducting Si Nanowire Arrays. J. Appl. Phys. 2004, 96, 5921 10.1063/1.1801155.

[ref56] Palsdottir T.; Nordstrom T.; Karlsson A.; Grönberg H.; Clements M.; Eklund M. The Impact of Different Prostate-Specific Antigen (PSA) Testing Intervals on Gleason Score at Diagnosis and the Risk of Experiencing False-Positive Biopsy Recommendations: A Population-Based Cohort Study. BMJ. Open 2019, 9, e027958 10.1136/bmjopen-2018-027958. PMC6475177 30928965

[ref57] Esteban E. P.; Almodovar-Abreu L. A New Interpretation of the Standard PSA-Test. Res. Reports Urol. 2020, 12, 75–84. 10.2147/RRU.S240171. PMC7060028 32185150

[ref58] Zhang D.; Lu Y.; Zhang Q.; Liu L.; Li S.; Yao Y.; Jiang J.; Liu G. L.; Liu Q. Protein Detecting with Smartphone-Controlled Electrochemical Impedance Spectroscopy for Point-of-Care Applications. Sensors Actuators B Chem. 2016, 222, 994–1002. 10.1016/j.snb.2015.09.041.

[ref59] Goh C. M.; Meriaudeau F.; Saad N. M.; Subramaniam R.; Ali S. A. Subcutaneous Veins Depth Measurement Using Diffuse Reflectance Images. Opt. Express 2017, 25, 25741–25759. 10.1364/OE.25.025741. 29041239

[ref60] Oltulu P.; Ince B.; Kokbudak N.; Findik S.; Kilinc F. Measurement of Epidermis, Dermis, and Total Skin Thicknesses from Six Different Body Regions with a New Ethical Histometric Technique. Turkish J. Plast. Surg. 2018, 26, 56 10.4103/tjps.TJPS_2_17.

[ref61] Gill H. S.; Denson D. D.; Burris B. A.; Prausnitz M. R. Effect of Microneedle Design on Pain in Human Subjects. Clin. J. Pain 2008, 24, 585 10.1097/AJP.0b013e31816778f9. 18716497 PMC2917250

[ref62] Mohammed B. S.; Cameron G. A.; Cameron L.; Hawksworth G. H.; Helms P. J.; McLay J. S. Can Finger-Prick Sampling Replace Venous Sampling to Determine the Pharmacokinetic Profile of Oral Paracetamol?. Br. J. Clin. Pharmacol. 2010, 70, 52–56. 10.1111/j.1365-2125.2010.03668.x. 20642547 PMC2909807

[ref63] Azzouzi A.-R.; Larre S.; Cormier L.; Roupret M.; Valeri A.; Mangin P.; Berthon P.; Villette J.-M.; Fiet J.; Cussenot O. Relevance of the Prostate-Specific Antigen (PSA) Nanotest Compared to the Classical PSA Test in the Organized Mass Screening of Prostate Cancer. BJU Int. 2007, 99, 762–764. 10.1111/j.1464-410X.2006.06701.x. 17233806

[ref64] Miano R.; Mele G. O.; Germani S.; Bove P.; Sansalone S.; Pugliese P. F.; Micali F. Evaluation of a New, Rapid, Qualitative, One-Step PSA Test for Prostate Cancer Screening: The PSA RapidScreen Test. Prostate Cancer Prostatic Dis. 2005 83 2005, 8, 219–223. 10.1038/sj.pcan.4500802. 15897915

[ref65] Aellen J.; Dabiri A.; Heim A.; Liaudet L.; Burnier M.; Ruiz J.; Feihl F.; Waeber B. Preserved Capillary Density of Dorsal Finger Skin in Treated Hypertensive Patients with or without Type 2 Diabetes. Microcirculation 2012, 19, 554–562. 10.1111/j.1549-8719.2012.00188.x. 22578093

[ref66] Debbabi H.; Uzan L.; Mourad J. J.; Safar M.; Levy B. I.; Tibiriçà E. Increased Skin Capillary Density in Treated Essential Hypertensive Patients. Am. J. Hypertens. 2006, 19, 477–483. 10.1016/j.amjhyper.2005.10.021. 16647618

[ref67] Houben A. J. H. M.; Martens R. J. H.; Stehouwer C. D. A. Assessing Microvascular Function in Humans from a Chronic Disease Perspective. J. Am. Soc. Nephrol. 2017, 28, 3461–3472. 10.1681/ASN.2017020157. 28904002 PMC5698072

[ref68] Monticone G.; Colonna L.; Palermi G.; Bono R.; Puddu P. Quantitative Nailfold Capillary Microscopy Findings in Patients with Acrocyanosis Compared with Patients Having Systemic Sclerosis and Control Subjects. J. Am. Acad. Dermatol. 2000, 42, 787–790. 10.1067/mjd.2000.103046. 10775855

[ref69] Tibiriçá E.; Rodrigues E.; Cobas R.; Gomes M. B. Increased Functional and Structural Skin Capillary Density in Type 1 Diabetes Patients with Vascular Complications. Diabetol. Metab. Syndr. 2009, 1, 24 10.1186/1758-5996-1-24. 19958533 PMC2794258

[ref70] Jiang S.; Li P.; Yu Y.; Liu J.; Yang Z. Experimental Study of Needle-Tissue Interaction Forces: Effect of Needle Geometries, Insertion Methods and Tissue Characteristics. J. Biomech. 2014, 47, 3344–3353. 10.1016/j.jbiomech.2014.08.007. 25169657

[ref71] Ranamukhaarachchi S. A.; Stoeber B. Determining the Factors Affecting Dynamic Insertion of Microneedles into Skin. Biomed. Microdevices 2019, 21, 100 10.1007/s10544-019-0449-y. 31745652

[ref72] Peled A.; Pevzner A.; Peretz Soroka H.; Patolsky F. Morphological and Chemical Stability of Silicon Nanostructures and Their Molecular Overlayers under Physiological Conditions: Towards Long-Term Implantable Nanoelectronic Biosensors. J. Nanobiotechnology 2014, 12, 7 10.1186/1477-3155-12-7. 24606762 PMC3975481

[ref73] Mejak S. L.; Bayliss J.; Hanks S. D. Long Distance Bicycle Riding Causes Prostate-Specific Antigen to Increase in Men Aged 50 Years and Over. PLoS One 2013, 8, e56030 10.1371/journal.pone.0056030. 23418500 PMC3572135

[ref74] Jiandani D.; Randhawa A.; Brown R. E.; Hamilton R.; Matthew A. G.; Kuk J. L.; Alibhai S. M. H.; Tufts E.; Santa Mina D. The Effect of Bicycling on PSA Levels: A Systematic Review and Meta-Analysis. Prostate Cancer Prostatic Dis 2015, 18, 208–212. 10.1038/pcan.2015.16. 25939515

[ref75] Mashkoor F. C.; Al-Asadi J. N.; Al-Naama L. M. Serum Level of Prostate-Specific Antigen (PSA) in Women with Breast Cancer. Cancer Epidemiol 2013, 37, 613–618. 10.1016/j.canep.2013.06.009. 23932967

