# Supplementary material for: The “Bloodless” Blood Test: Intradermal Prick Nanoelectronics for the Blood Extraction-Free Multiplex Detection of Protein Biomarkers
Source: ACS Nano. 2022 Aug 25;16(9):13800–13. doi: 10.1021/acsnano.2c01793 (PMC9527802; doi:10.1021/acsnano.2c01793)
Supplement: Supplementary file 1 — nn2c01793_si_001.pdf [file nn2c01793_si_001.pdf]

Supporting information

The 'Bloodless' Blood Test: Intradermal Prick Nanoelectronics for the Blood Extraction-free Multiplex Detection of Protein Biomarkers

Nimrod Harpak[1#], Ella Borberg[1#], Adva Raz[2] and Fernando Patolsky[1,2*]

1. School of Chemistry, Faculty of Exact Sciences, Tel Aviv University, Tel Aviv, 69978, Israel.
2. Department of Materials Science and Engineering, the Iby and Aladar Fleischman Faculty of Engineering, Tel Aviv University, Tel Aviv 69978, Israel.

# Authors contributed equally.

Emails: fernando@post.tau.ac.il

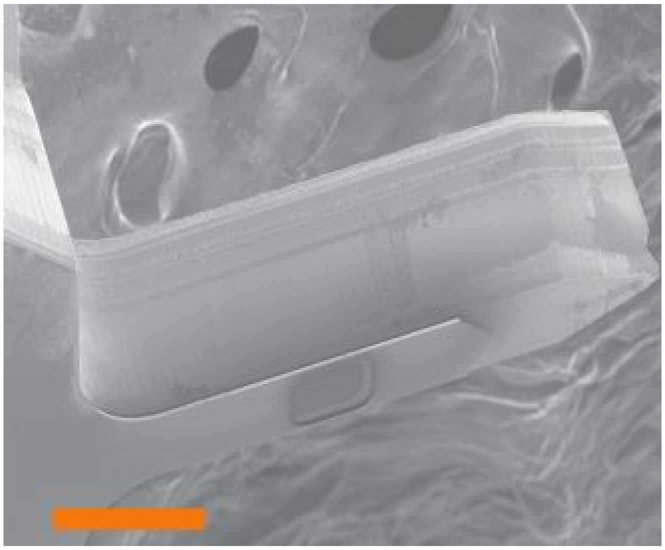

Figure S1 | Scanning electron microscopy image of a microneedle tilted view, scale bar: 250 μm.

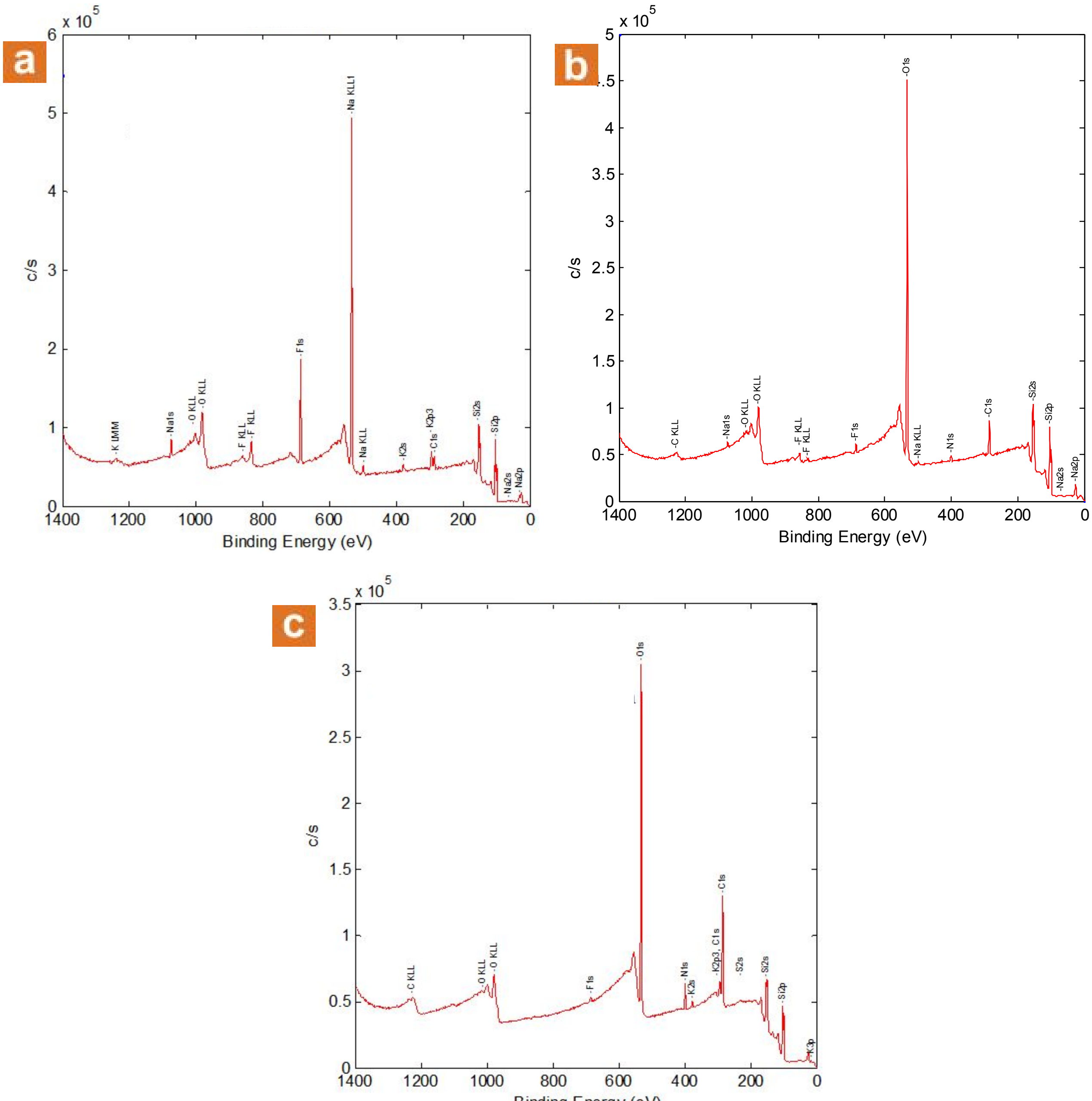

Z

Figure S2 | XPS characterization of modification steps. **(a)** Clean silicon wafer. **(b)** wafer modifies with amino-silane. **(c)** wafer modifies with IgG.

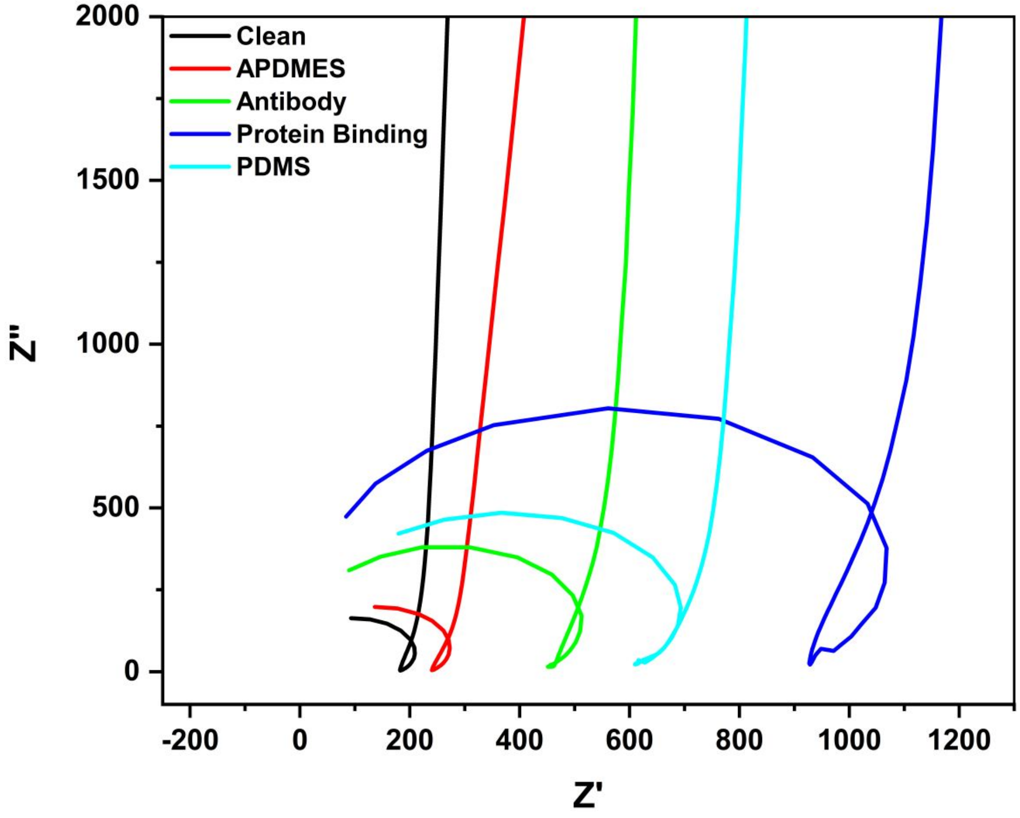

Figure S3 | Electrochemical impedance spectroscopy of the modification steps. Measurements were performed using a three-electrode system (silicon piece as working electrode, platinum mesh as counter electrode and Ag/AgCl as a reference electrode) submerged in 0.01X PBS solution, under 20mV amplitude at 0.1V.

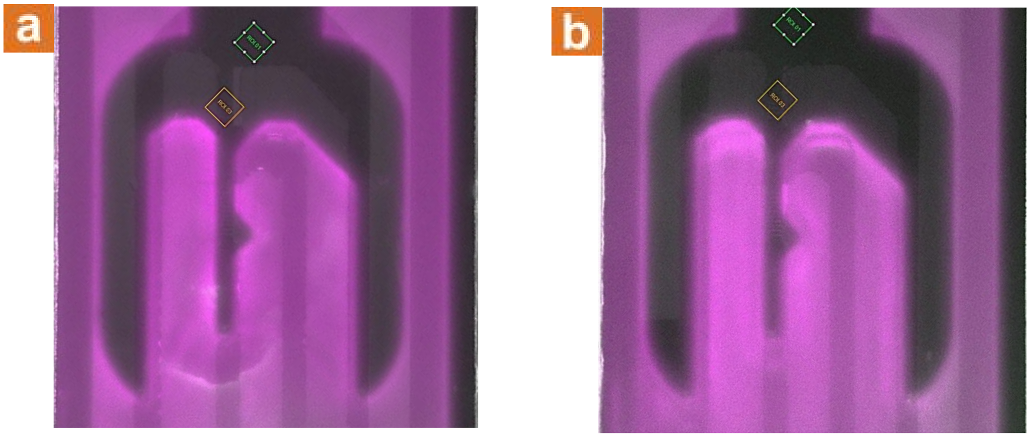

Figure S4 | Fluorescence microscopy images of Alexa488 chemically immobilized to needles with SU8 window before **(a)** and after **(b)** insertion to PDMS.

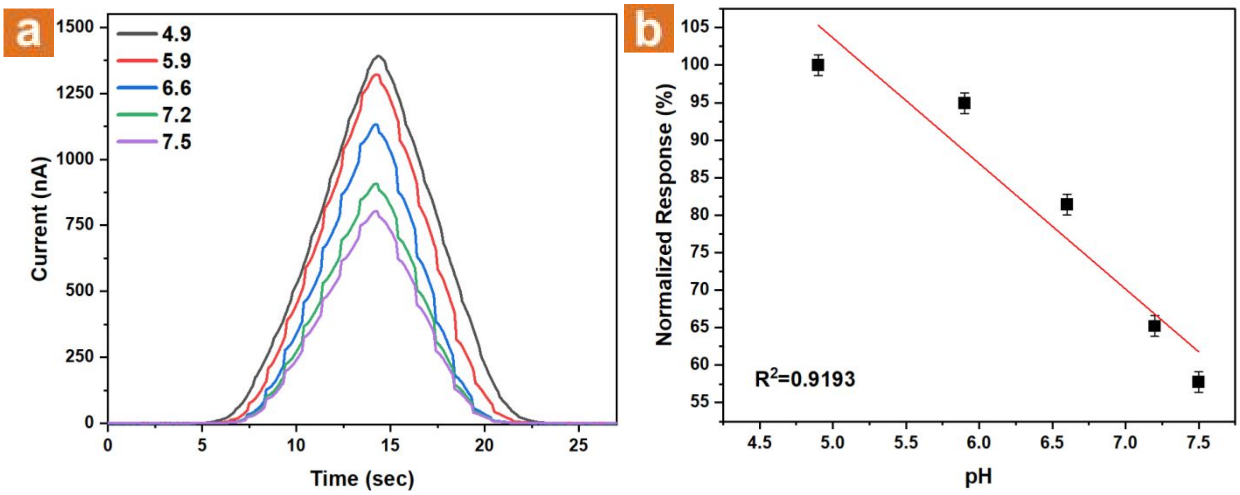

Figure S5 | Electrical pH measurements using the microneedle array without fluidic devices. (**a)** One cycle close-up view of the electrical measurement results of measurements in solutions of pH 4.9-7.5. The microneedles were dipped in 2ml solution of each pH for 5-10 minutes until stabilization. **(b)** Relative change in signal in comparison to pH 4.9. The colors in the graph represent the same pH values as in **(a)**.

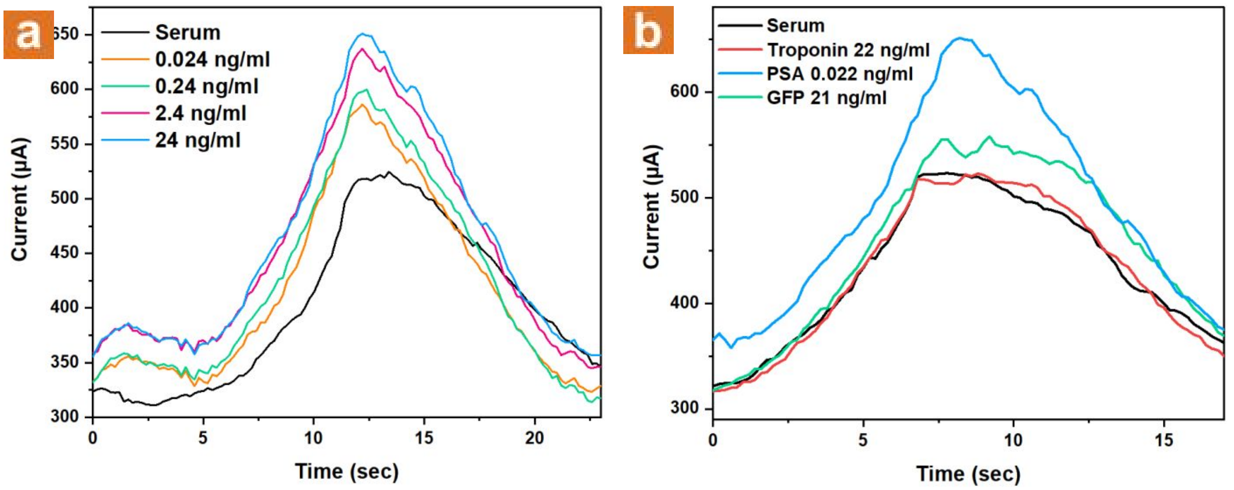

Figure S6 | Normalized electrical measurements of PSA-spiked serum and specificity measurements. **(a)** One cycle close-up view of the electrical measurement results of dissociation stabilization of the non-spiked serum (black curve), and serum spiked with 0.024-24 ng/ml PSA (orange to blue curves). **(b)** One cycle close-up view of the electrical measurement results of serum (black curve), 21 ng/ml GFP-spiked PBS solution (green curve), 22 ng/ml cTnI-spiked PBS solution (red curve), and 0.022 ng/ml PSA-spiked PBS solution (blue curve). The dissociation phase was conducted in 5% EG in 100μM phosphate buffer solution.

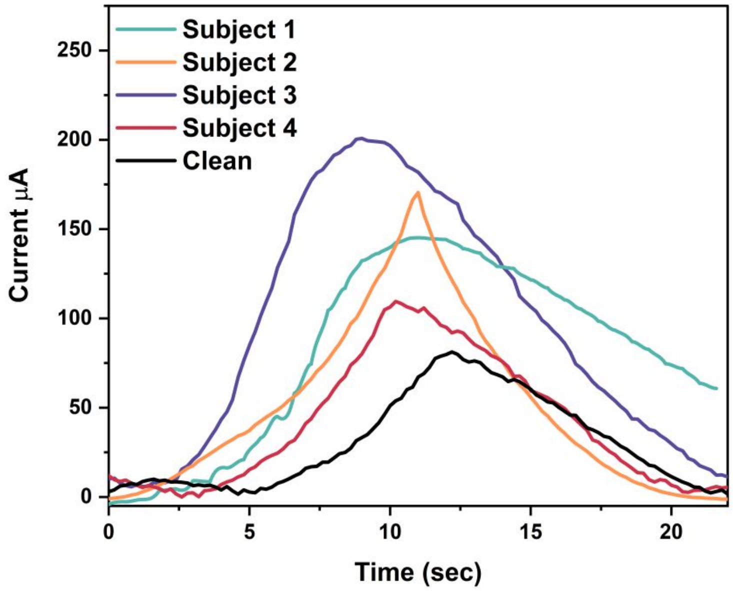

Figure S7 | One cycle close-up view taken once dissociation stabilization is achieved for clinical PSA measurements of four subjects. The dissociation phase was conducted in 5% EG in 100μM phosphate buffer solution. Clean refers to bovine serum.

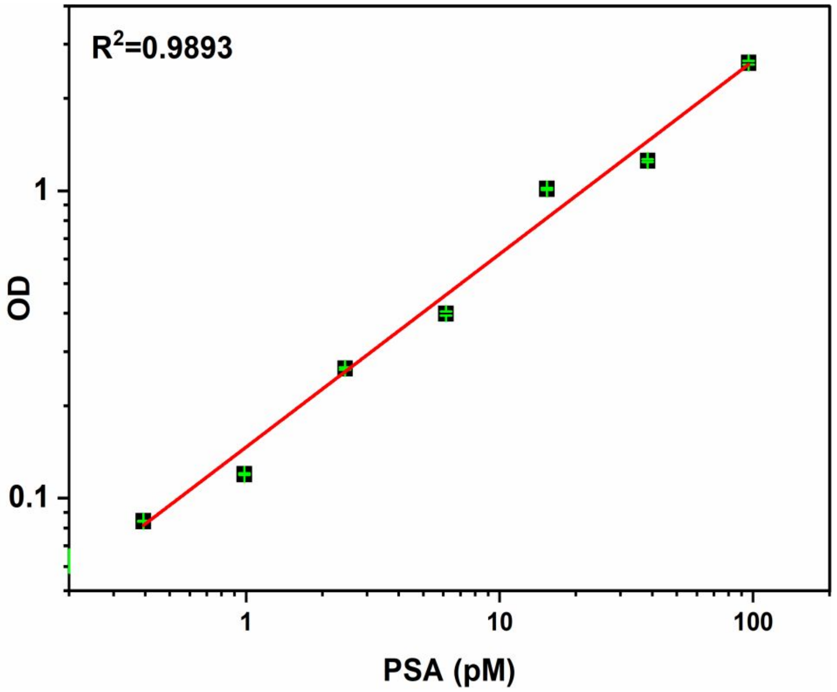

Figure S8 | Enzyme-linked immunosorbent assay calibration curve for PSA concentrations.

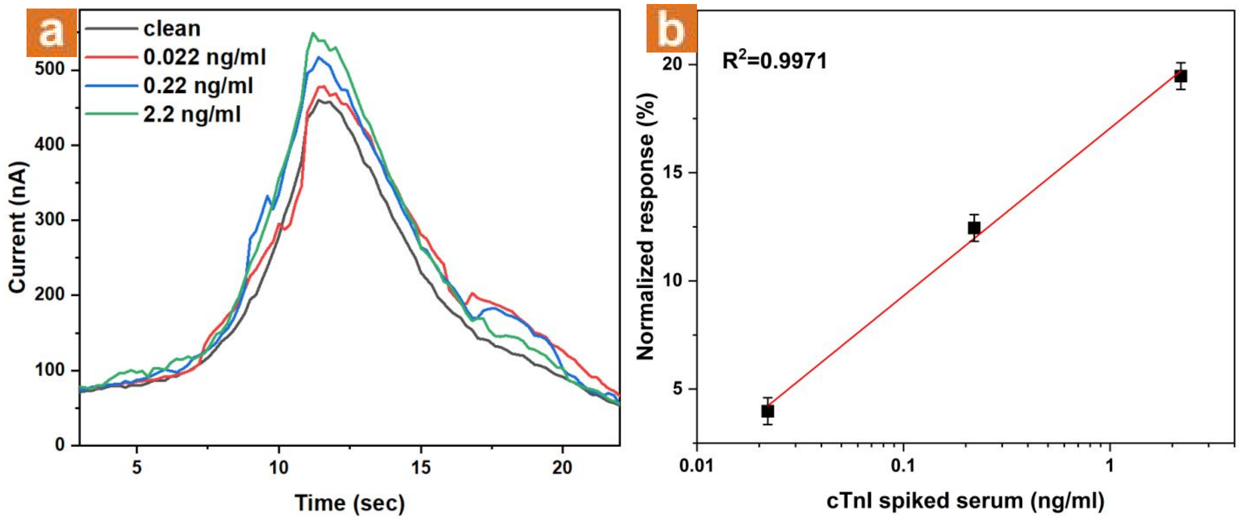

Figure S9 | Normalized electrical measurements of Troponin-spiked serum. **(a)** One cycle close-up view taken once dissociation stabilization is achieved for cTnI-spiked serum. **(b)** Response curve derived from **(a)**. The dissociation phase was conducted in 5% EG in 100μM phosphate buffer solution.

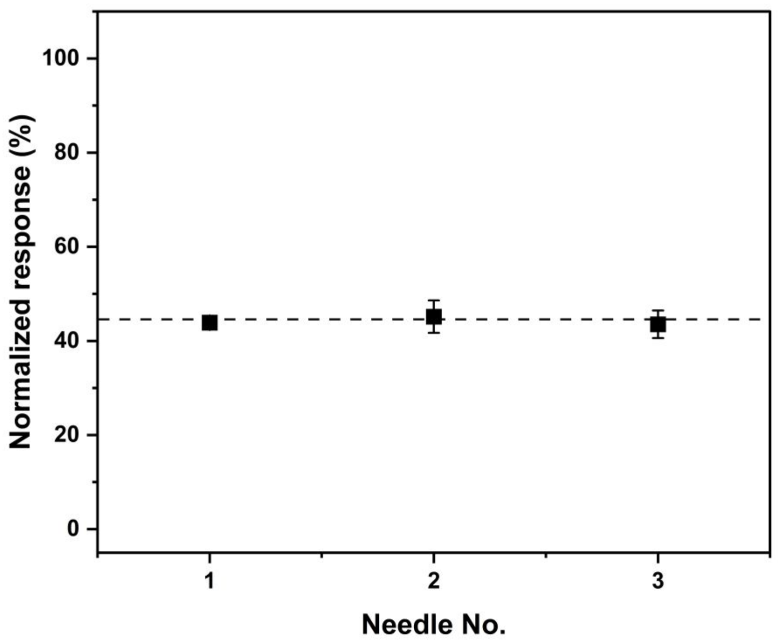

Figure S10 | Variation in normalized response between needles, derived from **Figure 5i.**
